# Information Fusion of Conflicting Input Data

**DOI:** 10.3390/s16111798

**Published:** 2016-10-29

**Authors:** Uwe Mönks, Helene Dörksen, Volker Lohweg, Michael Hübner

**Affiliations:** 1 inIT—Institute Industrial IT, Ostwestfalen-Lippe University of Applied Sciences, Lemgo 32657, Germany; helene.doerksen@hs-owl.de (H.D.); volker.lohweg@hs-owl.de (V.L.); 2 ESIT—Embedded Systems for Information Technology, Ruhr-University Bochum, Bochum 44801, Germany; michael.huebner@ruhr-uni-bochum.de

**Keywords:** information fusion, sensor fusion, conflict, evidence theory, Dempster-Shafer theory, possibility theory, fuzzy set theory

## Abstract

Sensors, and also actuators or external sources such as databases, serve as data sources in order to realise condition monitoring of industrial applications or the acquisition of characteristic parameters like production speed or reject rate. Modern facilities create such a large amount of complex data that a machine operator is unable to comprehend and process the information contained in the data. Thus, information fusion mechanisms gain increasing importance. Besides the management of large amounts of data, further challenges towards the fusion algorithms arise from epistemic uncertainties (incomplete knowledge) in the input signals as well as conflicts between them. These aspects must be considered during information processing to obtain reliable results, which are in accordance with the real world. The analysis of the scientific state of the art shows that current solutions fulfil said requirements at most only partly. This article proposes the multilayered information fusion system MACRO (multilayer attribute-based conflict-reducing observation) employing the *μ*BalTLCS (fuzzified balanced two-layer conflict solving) fusion algorithm to reduce the impact of conflicts on the fusion result. The performance of the contribution is shown by its evaluation in the scope of a machine condition monitoring application under laboratory conditions. Here, the MACRO system yields the best results compared to state-of-the-art fusion mechanisms. The utilised data is published and freely accessible.

## 1. Introduction

The basic *information fusion* (IFU) concept relies on the fact that data, which is supplied by sources, lacks information, which is completed in the fusion process. The common resulting effect is the generation of information, which is more dense and of higher quality than that of every single data source [1], and thus a decrease of the result’s inherent uncertainty. IFU can be carried out in two principle ways to achieve advantages over systems, which rely on a single source [2,3]:

**Definition 1** (Direct fusion). Combination of multiple sources of the same type to profit from statistical advantages due to an increased number of sources, and from redundancy effects compensating noisy or defective sources.

**Definition 2** (Indirect fusion). Combination of multiple sources of different types to incorporate information, which is available in some sources, but not in others, in the fusion process. Indirect fusion can be regarded as a generalisation of direct fusion.

Applications range from military (target tracking [2]) over industrial (fault diagnosis [4]) up to private (evaluation of vital data by members of the Quantified Self movement [5]). Systems to consider in this context are, among others, personal fitness trackers, smartphones, computation platforms in general (*system on chip* (SoC)), or transportation systems (cars, trains). They integrate more and more features, which have previously been served by other dedicated systems. Then more data sources are available in the integrated system on the one hand, but on the other hand the whole system becomes more complex. This situation is aggravated as such systems typically work in a variable environment and/or on a non-stationery process. Such variations result from varying qualities of the input components or changes in the environment (temperature, humidity, etc.), but also from ageing effects of the system and its components. Variations are nevertheless allowed, such that a system will behave differently after some time, but the result is within a defined acceptable variation range.

In this article, systems from machine and plant engineering, which are equipped with technical sensor units as data sources, are considered in the following. The signals acquired from technical applications are always prone to imprecision and uncertainty. In addition, conflicts might occur between input signals. Conflicts occur each time the information of one or more sources is not in line with the information of at least one other source. Such conflicts must be considered to derive the correct decision. For reliable operation of these systems, continuous system state determination and assessment (condition monitoring) is necessary. The system’s sensors continuously measure the physical quantities, which are necessary for system control and monitoring.

Human cognitive capacities overburden by the system’s complexity and variability. On the one hand, the number of possible system states cannot be captured by a human operator; deep and precise human understanding of the whole system can thus hardly be obtained, which on the other hand makes the identification and localisation of system faults and defects difficult. Here, the precise knowledge about the system’s state is demanded, but not available.

One possible solution to tackle this situation is an automated approach, which makes use of the data available in the system to determine and assess its state. Therefore, this article presents the *multilayer attribute-based conflict-reducing observation* (MACRO) information fusion system, which operates in conflicting as well as conflict-free situations. Its core fusion algorithm *fuzzified balanced two-layer conflict solving* (*μ*BalTLCS) determines and handles conflicts between input signals, such that the conflicts’ effects on the fusion result are decreased.

In order to incorporate arbitrary data sources in the fusion process, they need to be available in a comparable form. This is achieved by acquiring information from the captured data through extracting expressive features. This information is then transformed into a coherent space by an information processing system. Methods applied to fulfil this task are located in the fields of *probability theory* (ProbT) [6,7,8], *Dempster-Shafer theory of evidence* (DST) [9,10], *fuzzy set theory* (FST) [11], and *possibility theory* (PosT) [12]. If necessary, the information is transferred from one theory to another.

Each theory is capable of modelling uncertainties connected with the information to be fused. Uncertainty is categorised into two types, aleatory and epistemic uncertainty [13,14]. When the data is complete and of other than deterministic nature (thus random/stochastic), its inherent uncertainty is referred to as aleatory. Epistemic uncertainty arises from ignorance resulting from incomplete knowledge about the system or process. While modelling epistemic uncertainty by means of probability theory is not appropriate, evidence theory-based models are instead more suitable.

Besides uncertainty modelling, the handling of conflicts between input sources is another crucial aspect. Neither ProbT, nor FST, nor PosT deliver appropriate means. Only DST considers conflict by its intrinsic rule of combination, but it has certain deficiencies in high-conflicting situations [15,16].

The theoretical background and applications of MACRO and *μ*BalTLCS have been researched over the past years and published in several papers [17,18,19,20,21,22,23,24]. This article summarises the research to present a comprehensive view on MACRO and *μ*BalTLCS. For further information, the interested reader is referred to [25].

The article is structured as follows. Related work is presented in the next section. The details on *multilayer attribute-based conflict-reducing observation* (MACRO) and *fuzzified balanced two-layer conflict solving* (*μ*BalTLCS), along with their constituent parts, follow in Section 3. Section 4 evaluates the presented methods in the scope of a machine condition monitoring application under laboratory conditions. Its results are discussed in Section 5. The article concludes with Section 6.

## 2. Related Work

Information fusion has been researched since more than 40 years, is scientifically well understood, but is still a very active field of research. The concept of IFU is to create new or more precise knowledge about physical quantities, events, situations , etc. by the utilisation of different information sources. With respect to technical systems, IFU has gained more attention starting in the 1970s when new sensors, advanced processing techniques, and increasingly powerful processing hardware became available. From then, data processing models and fusion algorithms have been driven nearly exclusively by applications in the military defence sector. During the 1990s and early 2000s, those algorithms have been adopted by the civil sector for usage in industrial fault diagnosis and condition monitoring applications [2].

After [26], the combined performance $\mathrm{Perf}(S_{1}\cup S_{2})$ of two sensors $S_{1}$ and $S_{2}$, which work with complementary physical principles, regarding the chosen criterion will be increased by IFU, such that

$$\mathrm{Perf}\left(S_{1}\cup S_{2}\right)>\mathrm{Perf}\left(S_{1}\right)+\mathrm{Perf}\left(S_{2}\right),\;\mathrm{or}\mathrm{at}\mathrm{least}:\;\mathrm{Perf}\left(S_{1}\cup S_{2}\right)>\max\left(\mathrm{Perf}\left(S_{1}\right),\mathrm{Perf}\left(S_{2}\right)\right).$$

This property, indicating advantages of integration of multiple information sources compared to the utilisation of a single source, is also observed in neurological examinations. All living biological systems constantly make intuitive and subconscious use of IFU, which protects themselves from danger and guarantees survival [27,28]. Mercier et al. showed that humans react slower towards an external stimulus acquired by one sense (audio or visual) than if these stimuli appear combined (audio-visual) [29]. They explain this effect with the *redundant target effect* (RTE) [29,30], which describes that neurons are activated to information acquired by multiple senses before every single information would have caused a separate activation (*coactivation*) [31]. Triggered by [30], Tozzi and Peters transferred these findings to the area of *algebraic topology* and relate the observed effect to the *Borsuk-Ulam theorem* (Satz II [32]) in [33]. The Borsuk-Ulam theorem expresses that any *antipodal* points on an *n*-dimensional sphere (examples of antipodal points are the poles of the Earth or exactly opposite points on a circle) are projected onto one point when the sphere is projected to an *n*-dimensional Euclidean space. According to Tozzi and Peters, the audio and visual sense represent the antipodal points on the sphere, which are both stimulated by the same event (their Euclidean projection). Hence, both share information about the event in their respective stimuli [33,34,35]. The full information of a stimulus is thus only observable by the combination (*fusion*) of multiple senses, otherwise parts of the information remain hidden resulting in incomplete knowledge.

The interested reader is referred to literature [2,36,37,38] for further information on basic principles of IFU and their applications. Comprehensive studies on contemporary research on IFU are found in [39,40]. Khaleghi et al. identify in their review article a number of main challenges posed on IFU systems arising from their input data. These are data imperfection (like uncertainty), outliers and spurious data, conflicting data, data modality, data correlation, data alignment/registration, data association, processing framework, operational timing, static vs. dynamic phenomena, and data dimensionality. No IFU approach, which addresses all of the aforementioned challenges is available [39].

Today, research knowledge is mutually transferred between all aforementioned application areas. Recent research regarding IFU—besides ongoing military research—is carried out in network traffic modelling scenarios [41], in the home care sector (*ambient assisted living* (AAL)) [42], as well as in the industrial context (machine diagnosis) [43]. Isermann provides a comprehensive introduction for an important application field of IFU: fault diagnosis of dynamic technical systems, mainly from a control theoretical point of view for process automation and in the automotive area (driver-assistance systems, autonomous driving, etc.). He provides a taxonomy for fault diagnosis systems and related areas, describes the advantages which can be obtained by fault diagnosis, discusses the relevant approaches, and illustrates a number of applications in this field [4,43]. Other applications contain condition monitoring of rotating electrical machines [44,45], electrical power supplies [46,47], intelligent transportation systems [48,49], or communication networks [50,51].

All of these information fusion areas are affected by conflicting inputs and data uncertainty. These aspects are discussed in the following.

### 2.1. Conflict

Whenever the information of at least one source disagrees with the remaining available information, conflict occurs. The possible causes of conflict are numerous. Source deterioration or faults occur especially in real-world problems. Manipulation of the sources (or their information) is also conceivable, especially in security-critical settings. Conflict is formally a form of conscious ignorance. It is namely the cause of *inconsistency* or distorted information [13]. Such information inconsistencies lead to results, which do not represent the actual situation, if the conflict has not been recognised and addressed during information processing. This also applies to humans [52]. Grüeninger et al. investigate museum visitors’ reflections of exhibition samples, where information about the samples conflicts. The inconsistency was recognised by more than 90% of the visitors, but less than 70% processed it.

Conflict has been identified as one of the most challenging topics in IFU by Khaleghi et al. [39]. Measures of conflict are well-known in literature. One example is Shannon’s *entropy measure* (defined as a measure of information [53]), which can also serve as a conflict measure [13].

A number of publications work on the improvement or substitution of the conflict quantified and processed in the combination rule of *Dempster-Shafer theory of evidence* (DST) [9,10]. Martin et al. propose a conflict measure based on the distance between belief functions. This new measure additionally serves to determine *a posteriori* the reliability of the recently processed data [54,55]. Smarandache et al. put this new approach into context and benchmark it against other conflict measures (which they call “contradiction measures”) [56]. A measure based on vector distances between the data to be fused is introduced in [57]. Other works include [58,59,60].

To a certain extent, conflict handling is independent from the model applied to represent the information. Whereas *probability theory* (ProbT) [6,7,8], *fuzzy set theory* (FST) [11], and *possibility theory* (PosT) [12] need to incorporate further processing steps for conflict handling, DST is inherently designed to handle conflicts: its fusion operation *Dempster’s rule of combination* (DRC) includes a term in the denominator, which is necessary for normalisation ([10], p. 60). This normalisation factor is interpreted as a measure of the extent of conflict between two beliefs [10]. The conflict’s extent is quantified ranging from 0 in the case of no conflict to 1 in the case of maximum conflict. DST’s core concepts of basic belief assignment as well as *belief* and *plausibility* functions are widely accepted in the scientific community. However, Dempster’s rule of combination has been discussed controversially almost right from the beginning. Criticism and research on alternatives compensating identified deficiencies is found in literature up to date [61,62,63,64]. The first discussion was raised by Zadeh and made public in 1984 [65], although the argumentation was already recorded in a technical report from 1979 [66]: Due to the normalisation applied in DRC, the combination result is counterintuitive in conflicting situations, in which experts are confident a certain proposition does not exist. He illustrates this situation with the following case of two physicians examining a patient, which in the literature is denoted by “Zadeh’s example”.

**Example 1** (Zadeh’s example). Two physicians are asked to assess a patient’s disease. Each expresses their belief as presented in Table 1.

Thus, either physician certainly rejects one of the three possible diseases and believes in brain tumor to only 1%.

Applying DRC will lead to the conclusion that the patient has a brain tumor with 100% belief (Table 2).

Zadeh hence argues that DRC yields counterintuitive fusion results in such a high-conflicting setting, as brain tumor has been excluded almost completely by both doctors [65]. His conclusion is supported by the “Real Z-box Experiment” of Dezert et al. [67].

Other authors discovered deficiencies of DRC similar to Zadeh’s findings (cf. [16,68]), which led to a number of alternative combination rules [39]. Murphy’s rule computes the arithmetic mean of the masses [69]. Yager’s alternative distributes the conflicting belief among all elements rather than only among the focal elements [61]. Campos’ rule renormalises the initial DRC result with respect to the conflict and thus avoids counterintuitive fusion results [70]. Dubois and Prade introduced a combination rule, which assigns conflicting mass to their focal elements’ union [71].

Other research defends DRC and argues that counterintuitive results occur due to improper application of DRC. Haenni argues that a concept should not be abandoned because it does not yield the desired result in a special situation. He furthermore underlines DRC’s validity by following Sherlock Holmes’ argumentation: something must be true, even if it is improbable, when all other alternatives turn out to be impossible [72,73]. Compared to Zadeh’s example the latter argumentation is not valid as each of the alternatives is possible as at least one doctor assigns them belief, i.e., none of the alternatives has assigned zero belief considering it completely impossible. Haenni also invalidates Zadeh.1984’s example by pointing out that Zadeh applied DST incorrectly by limiting the frame of discernment to only the given three diseases. His argument is instead that the frame of discernment must be augmented with combinations of the diseases as these are not mutually exclusive [73]. This argumentation depends on the semantics of the application and is thus not generally acceptable. That is, while the frame of discernment defined in Zadeh’s example is justifiably inappropriate, applications in which three mutually exclusive alternatives form the frame of discernment may exist. The observed counterintuitivity of DRC will be present in such cases. Apart from replacing the combination rule, Mahler votes for a transformation of the input data [74]. As is pointed out in [39], Mahler argues that the assignment of an arbitrary small non-zero mass to every proposition will circumvent counterintuitive results.

Another important aspect to be considered in IFU is uncertainty, which is content of the next section.

### 2.2. Uncertainty

Classification of uncertainty leads to two major types [13]:

**Definition 3** (Aleatory uncertainty). Aleatory uncertainty is characterised by its random and non-deterministic nature and thus represents the inherent randomness of a problem.

**Definition 4** (Epistemic uncertainty). Epistemic uncertainty is also denoted by subjective uncertainty. Its source is the lack of knowledge due to, e.g., incomplete data.

Klir and Wierman point out that uncertainty mostly cannot be avoided, especially in the context of real-world applications [75]. In engineering, uncertainty is caused by deficiencies in the acquisition of knowledge like measurement errors, lack of repetitions of an experiment, or production tolerances [14]. However, uncertainty can be kept to a minimum with the necessary information available. This applies to epistemic uncertainty. Aleatory uncertainty is due to its pure random character irreducible, but can be modelled.

Ayyub and Klir propose the framework of ProbT in case uncertainty is quantifiable [13]. However, ProbT is applicable to model information, which is affected by aleatory uncertainty, as probability distribution by a *probability density function* (pdf). Thus, Ayyub and Klir admit that epistemic uncertainty, which is the most dominant uncertainty type in risk analysis, can only be modelled with additional effort as a probabilistic variable. In addition, all methods based on Bayes’ theorem necessarily assume that data is acquired from independent sources, whose statistical behaviour is identical [6]. Independence must be questioned in most real-world applications, as data sources used in the same applications are at best partly decorrelated [76,77]. In addition, the prior must be determined before any application, whereas statistical information is not available for every application [78]. As a last resort, assumptions have to be made resulting in the prior to be uniform (in case of total ignorance) or Gaussian (due to its properties) [79].

Another possibility to model uncertain information is given by Pawlak’s *rough set theory* [80]. It deals with incomplete information by approximation of crisp sets. Each set is represented by a tuple of sets, of which one is the lower and one the upper approximation. The lower approximation contains all elements, which are definitely member of the set, while the upper approximation contains elements which possibly belong to the set. Fusion is carried out by classic set operations like conjunction and disjunction [81].

With respect to situations that are prevalent in the environment of machine and plant engineering (e.g., production processes [4]), uncertainty is mainly of epistemic nature. Here, only a subset of necessary knowledge for the precise assessment is available. The uncertainty is recognised, but cannot be expressed in statistical, hence probabilistic terms.

Therefore, this article presents a fusion approach, which is able to handle conflict and model epistemic uncertainties. This approach is elaborated in the following.

## 3. Multilayer Attribute-Based Conflict-Reducing Observation

This section introduces the *multilayer attribute-based conflict-reducing observation* (MACRO) information fusion system. It utilises a two-layer fusion system structure to resemble the physical structure of a monitored system. Conflict handling is implemented in the fusion operation denoted by *fuzzified balanced two-layer conflict solving* (*μ*BalTLCS), which is applied on MACRO’s lower layer. The amount of conflict between the inputs is determined and handled such that the conflict effects on the fusion result are reduced. MACRO’s final output is created by the information fusion operation on the top layer. It determines and assesses the current system state based on the fusion results of *μ*BalTLCS on the lower layer.

### 3.1. Architecture

The architecture of the MACRO system is designed to resemble the actual structure of the monitored system, which is partitioned into several sub-systems. This kind of architecture is found in contemporary system design of several application fields.

**Figure 1:**
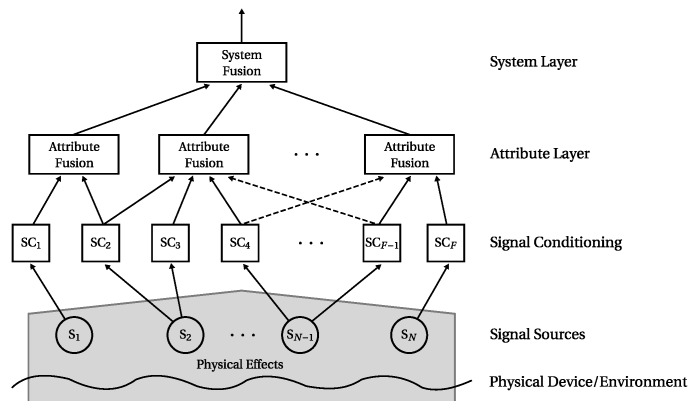
Architecture of the multilayer attribute-based conflict-reducing observation system MACRO [20].

The purpose of the MACRO fusion system (depicted in Figure 1) is to determine and assess the state of a complete system by monitoring its sub-systems and properties.

The monitoring is carried out as follows: Signals from the system as well as from its environment (like temperature) are acquired by sensors (*signal sources*). Features are extracted from the signals in the following *signal conditioning* step, which may also include signal preprocessing procedures. Here, one or multiple features may be extracted from one signal, e.g., to determine the mean and variance from one signal. MACRO then combines ensembles of conditioned signals in groups denoted by *attributes*. They represent certain properties or physical parts of the observed system. Hence, each attribute has a semantic meaning, which relates it to the physical system. An attribute’s output indicates to which degree its inputs represent the system’s normal condition and is denoted by *attribute health*. The attributes are application-dependent and manually defined during the fusion system design process. Redundancies, which occur by combining at least two information sources to one attribute, are exploited for both (i) detecting sensor faults and (ii) cross-checking the consistency of sensor values.

All attribute healths are fused on *system layer* by the *system fusion* algorithm. It determines and assesses the current system state denoted by *system health*. The system health is MACRO’s final output and indicates to which degree the system represents normal condition.

The basic multilayer structure of MACRO is also inspired by the decision-making process of groups of humans: Individual humans (sensors) discuss their opinions (measurements) in groups (attribute layer). This group decision-making process includes conflicts. The information generated in the various groups is then combined on organisational level (system layer) to make a global decision. For more information on the human group decision-making background of MACRO the reader is referred to [19].

This article concentrates on conflict-reducing fusion on MACRO’s attribute layer, whereas sensor fault detection is not in the scope of this article. A proposition for fault detection in the context of MACRO is given in [22]. The following section details the fusion algorithm used to mitigate the influence of conflicts between the inputs and to implicitly cross-check sensor value consistencies.

### 3.2. Balanced Two-Layer Conflict Solving

No matter whether DST’s original *Dempster’s rule of combination* (DRC) or other ad-hoc fusion rules are applied, none of them have been regarded as a superior method compared to others. This section proposes the *balanced two-layer conflict solving* (BalTLCS) fusion rule, which improves the fusion results in cases of high-conflict between input sensors. It employs findings from psychological research on human group decision-making and transfers them to the field of information fusion.

State-of-the-art psychological research reveals that human group decision-making is carried out effectively by (i) information exchange between individuals prior to (ii) decision on group level [82,83,84]. Following these psychological principles in information fusion yields a human-oriented approach leading to fusion results, in which the conflict between input information is processed. These two principles are incorporated in the *Two-Layer Conflict Solving* (TLCS) fusion approach by Li and Lohweg. It involves pairwise fusion in *Conflict-Modified-DST* (CMDST) and subsequent fusion on group level facilitated by *Group-Conflict-Redistribution* (GCR). This DST-based approach effectively decreases the effect of conflicts on the fusion result [16]. However, the analysis of TLCS reveals a number of deficiencies [17]. Depending on the amount of conflict, TLCS yields counterintuitive fusion results. Its conflict measures yield no normalised results, making the involvement of a renormalisation factor in GCR necessary.

The fusion algorithm proposed in this section is on the one hand based on TLCS to exploit its positive properties, whereas the identified deficiencies of TLCS (especially its counterintuitivity) are mitigated on the other hand. This approach is denoted by BalTLCS and offers the following properties:

- adoption of effective human group decision-making principles,
- determination of conflicts between inputs,
- solution of the conflicts, such that their effect on the fusion result is decreased,
- creation of intuitive fusion results, also in high-conflict cases.

BalTLCS is like TLCS based on DST [9,10]. This theory works on a finite sample space called *frame of discernment* forming a set $\Theta =\left\{A_{1},A_{2},\cdots ,A_{n}\right\}$, where $A_{i}$ denotes a proposition or hypothesis. The power set $2^{\Theta }$ includes all possible combinations of propositions $A_{i}$. Propositions are regarded to be mutually exclusive and exhaustive. Thus, the power set contains $2^{n}$ subsets. The complete belief may be partitioned among the different subsets A⊆Θ. This degree of belief in proposition *A* is expressed by the *basic belief assignment* (BBA) m(A) defined as [10]:

**Definition 5** (Basic belief assignment). *If $\Theta =\left\{A_{1},A_{2},\cdots ,A_{n}\right\}$ is a frame of discernment, then a function $m:2^{\Theta }\to \left[0,1\right]$ is called basic belief assignment when*

$$m\left(⌀\right)=0\;\mathrm{and}\;\sum_{A\subseteq \Theta }m\left(A\right)=1.$$

The quantity m(A) is the individual belief assigned to *exactly* *A*, also denoted by *mass*. No mass is assigned the empty set ⌀, whereas the sum of all masses must be 1—in other words, not more than 100% of the individual belief may be assigned. Each subset of $2^{\Theta }$, which is assigned a nonzero basic belief, is called *focal element* of the frame of discernment.

BalTLCS determines intermediate fusion results with respect to non-conflicting and conflicting BBAs, which are subsequently combined. In this context, the limit cases of conflict between fusion inputs (sensors) are evaluated. These are:

**No** **conflict:** All sensors fully agree with *the same* proposition. Then the BBAs of all sensors for proposition $A_{i}$ are $m_{s}\left(A_{i}\right)=1$ for all *s*, whereas those for all other propositions $A_{j}$ are $m_{s}\left(A_{j}\right)=0$ for all *s* with j≠i.

**Maximum** **conflict:** All sensors fully agree with *a different* proposition. Each proposition $A_{i}$ is assigned a maximum BBA by sensor *s*, i.e., for all $t\neq s:m_{s}\left(A_{i}\right)=1$ and $m_{t}\left(A_{i}\right)=0$. All other propositions $A_{j}\neq A_{i}$ is assigned no BBA by sensor *s*: $m_{s}\left(A_{j}\right)=0$.

Each part of BalTLCS is detailed in the following subsections.

#### 3.2.1. Non-Conflicting Part

TLCS employs CMDST as a measure, which relates the non-conflicting belief in one proposition to the overall non-conflicting belief [16]. It thus represents an aggregated belief, which is purged from inherent conflict as only non-conflicting beliefs are involved. Based on this, the non-conflicting part for the fusion process of *n* sensors in the scope of BalTLCS is proposed as [19]:

**Definition 6** (BalTLCS: non-conflicting part). *The non-conflicting part of BalTLCS fusion is with n≥2 determined as:*

(1) $$m_{\mathrm{nc}}\left(A\right)=\frac{1}{\mathrm{Bc}(n)}\sum_{(s,t)\in S}m_{s}\left(A\right)\cdot m_{t}\left(A\right)=\frac{1}{\mathrm{Bc}(n)}\sum_{s=1}^{n-1}\sum_{t=s+1}^{n}m_{s}\left(A\right)\cdot m_{t}\left(A\right),$$

*where $S=\left\{\left(s,t\right)|s,t\in N_{n}=\left\{1,2,\cdots ,n\right\},s<t\right\}$ with S=Bc(n)=n2=12·n!(n-2)!=n2·(n-1). This set describes all possible combinations of the n sensors such that each sensor is combined once with the others.*

It is hence a measure, which relates the non-conflicting belief in proposition *A* to the maximally achievable non-conflicting belief in this proposition. The latter is achieved in the case of no conflict. Then $\sum_{(s,t)\in S}m_{s}\left(A\right)\cdot m_{t}\left(A\right)=\mathrm{Bc}(n)$ as $\left|S\right|=\mathrm{Bc}(n)$ combinations of two sensors are evaluated. The number of inputs *n* has a lower limit of n=2 as fusion of 1 or less inputs is physically infeasible.

The non-conflicting part of BalTLCS is a normalised measure with $0\leq m_{\mathrm{nc}}\left(A\right)\leq 1$, where $m_{\mathrm{nc}}\left(A\right)=1$ in the case of no conflict and $m_{\mathrm{nc}}\left(A\right)=0$ in the case of maximum conflict.

Conflicts between the sensors are determined and represented in the *conflicting part* of BalTLCS.

#### 3.2.2. Conflicting Part

The TLCS conflicting coefficient $k_{\mathrm{cm}}$ involves pairwise sensor combinations [16]. This principle of individual information exchange is preserved in BalTLCS. Whereas $k_{\mathrm{cm}}$ is not a normalised measure, BalTLCS proposes the normalised conflicting coefficient, which is interpretable as a degree of conflict [19]:

**Definition 7** (BalTLCS: normalised conflicting coefficient). *The degree of conflict between individual beliefs is modelled by the normalised conflicting coefficient c as:*

(2) $$\begin{array}{cc} c & =\frac{1}{\mathrm{Bc}(n)}\cdot k_{\mathrm{cm}}=\sum_{(s,t)\in M}\sum_{(i,j)\in A}m_{s}\left(A_{i}\right)\cdot m_{t}\left(A_{j}\right)=\frac{1}{\mathrm{Bc}(n)}\sum_{s=1}^{n-1}\sum_{t=s+1}^{n}\sum_{i=1}^{o}m_{s}\left(A_{i}\right)\cdot \left(1-m_{t}\left(A_{i}\right)\right)\\ & =1-\sum_{s=1}^{n-1}\sum_{t=s+1}^{n}\sum_{i=1}^{o}m_{s}\left(A_{i}\right)\cdot m_{t}\left(A_{i}\right)=1-\sum_{i=1}^{o}m_{\mathrm{nc}}\left(A_{i}\right), \end{array}$$

*where $A=\left\{\left(i,j\right)|i,j\in N_{o},i\neq j\right\}$. This set addresses the sensors’ beliefs about conflicting propositions.*

In the case of no conflict, the normalised conflicting coefficient yields c=0, whereas c=1 in the case of maximum conflict. Hence, 0≤c≤1.

The normalised conflicting coefficient is applied to control the conflicting part of BalTLCS [19]:

**Definition 8** (BalTLCS: conflicting part). *The conflicting part of BalTLCS fusion is determined as the arithmetic mean of all input BBAs weighed by c* [19]*:*

(3) $$m_{c}\left(A\right)=c\cdot \frac{1}{n}\sum_{s=1}^{n}m_{s}\left(A\right).$$

In the case of no conflict, the BalTLCS conflicting part yields $m_{c}\left(A\right)=0$, whereas $m_{c}\left(A\right)=\frac{1}{n}$ in the case of maximum conflict. Hence, $0\leq m_{c}\left(A\right)\leq \frac{1}{n}$. It leads to a balanced fusion result: none of the sensors are allowed to dominate the other, and none of them are allowed to influence the overall result with more than $n^{-1}$. Hence, the arithmetic mean determines the combined conflicting part supporting a certain proposition *A*, weighed with the degree of conflict *c*. This also ensures that the information content about the proposition is not shifted to the frame of discernment Θ (which defines ambiguity or ignorance) in a strong conflict case. Such is relevant especially in real-world applications, in which a decision must be made in all cases—also in high-conflict situations.

Both parts are subsequently combined by *balanced group conflict redistribution* (BalGCR), which is introduced in the following.

#### 3.2.3. Balanced Group Conflict Redistribution

In order to obtain the overall fusion result, the non-conflicting and conflicting parts of BalTLCS are connected in a subsequent additive fusion step [19]:

**Definition 9** (Balanced group conflict redistribution (BalGCR)). *Let $m_{\mathrm{nc}}\left(A\right)$ be the non-conflicting part of BalTLCS fusion after Equation (1) and $m_{c}\left(A\right)$ its conflicting part after Equation (3). Then the BalTLCS fusion result m(A) is determined by balanced group conflict redistribution (BalGCR) as*

(4) $$m\left(A\right)=m_{\mathrm{nc}}\left(A\right)+m_{c}\left(A\right).$$

Whereas the non-conflicting part is determined by pairwise aggregation, the conflicting part considers all sensors at the same time. Hence, BalGCR follows the same concept, which is applied in TLCS [16]: decision-making in the whole group employs the intermediate result which has been found in “bilateral discussions”, and the original BBAs of all sensors. In BalGCR, these two parts are additively connected.

The BBA assigned to the frame of discernment, which represents the amount of ignorance, follows directly from Definition 5. It is determined by

(5) $$m\left(\Theta \right)=1-\sum_{A_{i}\subset \Theta }m\left(A_{i}\right).$$

If no conflict occurs, the non-conflicting part determines the overall fusion result: $m\left(A\right)=m_{\mathrm{nc}}\left(A\right)$. If the conflict is maximal, then all information sources have to be taken into account, which is achieved by $m_{c}$ determining the arithmetic mean of all sensory hypotheses: $m\left(A\right)=\frac{1}{n}$. Thus, a balance between conflicting and non-conflicting beliefs is established by the additive connection applied in BalGCR utilising the conflicting coefficient *c* as a control parameter.

The necessary property, which results in m(A) being a BBA, is the boundedness of BalGCR’s output. This is shown by

**Lemma 1** (Boundedness of balanced group conflict redistribution). *Let $m(A_{i})$ be an aggregated BBA assigned to proposition $A_{i}$ obtained by BalGCR. Then the sum of all aggregated BBAs is*

$$\sum_{i=1}^{o}m\left(A_{i}\right)=1.$$

**Proof.**  See Appendix C. □

Due to this property, each $m(A_{i})$ satisfies Definition 5 and is therefore a *basic belief assignment*.

#### 3.2.4. Conclusions on Balanced Two-Layer Conflict Solving

The following conclusions are derived with respect to BalTLCS.

- BalTLCS fuses a number of input BBAs by determining intermediate results among the non-conflicting and the conflicting BBAs, and their subsequent additive combination by BalGCR.
- The non-conflicting part of BalTLCS fusion is determined by exhaustive individual combination of pairs of two sensors instead of combination of all sensors at the same time. This is inspired by psychological research findings on human group decision-making.
- In order to derive a decision in all cases, also in high-conflicting cases, the conflicting part is determined by the arithmetic mean amongst all sensors. This is additionally weighed by the normalised conflicting coefficient, such that the conflicting part plays only a subordinate role in the fusion process in case of no or small conflict.
- BalTLCS fusion yields intuitive results. This is evaluated and shown in [17].

After the introduction of the BalTLCS fusion approach, the following section introduces the quantity, which is observed in the scope of MACRO.

### 3.3. System State Representation

In order to determine the health of a system, optimally all possible system states must be known and quantifiable based on data delivered by the sensors. However, not all of the system’s operation points must necessarily be known to asses the system’s condition whether its behaviour is different from usual behaviour.

**Definition 10** (System condition). Due to experience, a machine operator is able to classify a system’s behaviour, although the assessment is in many cases not based on quantifiable perceptions. The following two classes of system conditions are at least distinguished.

${}^{N}C$:***normal-condition***. Nothing unusual or suspicious is perceived by the machine operator during system operation. The system fulfils its task as intended.

${}^{\bar{N}}C$:***abnormal-condition***. Unusual or suspicious effects are perceived by the machine operator during system operation. The system may or may not fulfil its task as intended.

This distinction between only said two conditions ${}^{N}C$ and ${}^{\bar{N}}C$ is utilised by MACRO. Uncertainties in the data acquired from mechanical and plant enigneering systems are of epistemic nature. Thus, the conditions are proposed to be modelled as standard fuzzy sets, one for each sensor $S_{s}$ and each condition:

**Definition 11**  (Standard fuzzy set [85]). *A fuzzy set A described by a membership function $\mu_{A}:\Theta \to \left[0,1\right]$ is called standard fuzzy set, if it is normal and convex, hence satisfies the following axioms* [11,85]:

**Axiom 1** (Normality). $\underset{\theta \in A}{sup}\;\mu_{A}\left(\theta \right)=1.$
**Axiom 2** (Convexity). $\mu_{A}\left(\lambda \theta_{1}+\left(1-\lambda \right)\theta_{2}\right)\geq min\left(\mu_{A}\left(\theta_{1}\right),\mu_{A}\left(\theta_{2}\right)\right),\forall \theta_{1},\theta_{2}\in \Theta ,\lambda \in \left[0,1\right]$.

Consequently, the universal set is $\Theta =\left\{{}^{N}C,{}^{\bar{N}}C\right\}$ in this case, where

- ${}^{N}\mu_{s}:R\to \left[0,1\right]$ models the normal condition,
- ${}^{\bar{N}}\mu_{s}:R\to \left[0,1\right]$ models the abnormal condition.

An experienced machine operator is able to determine whether the system is in normal condition or if the system’s behaviour suggests that it is not operating in normal condition. In the latter case, the system is consequently in abnormal condition ${}^{\bar{N}}C$, which is therefore the complement of ${}^{N}C$. The abnormal condition is proposed to be modelled by the *fuzzy standard complement* [85] as

(6) $${}^{\bar{N}}\mu_{s}=1-{}^{N}\mu_{s}.$$

Hence, the only relevant system condition is the normal condition. In order to derive a model of ${}^{N}C$, sensor data is acquired during the operation of the system, i.e., only data acquired by the sensors during system operation in normal condition is necessary to derive the model from. This data, which represents ${}^{N}C$, is applied to an automatic learning procedure to determine the membership function based on measurement data: Let the vector ${{}^{N}\theta }_{s}=\left({}^{N}\theta_{s}\left[1\right]{,}^{N}\theta_{s}\left[2\right],\cdots {,}^{N}\theta_{s}\left[N\right]\right)$ consist of *N* individual measurements ${}^{N}\theta_{s}\left[k\right]$ acquired by sensor $S_{s}$ during operation of a system under normal condition. Then the corresponding *normal condition membership function* ${}^{N}\mu_{s}$ is learned automatically following the *Modified-Fuzzy-Pattern-Classifier* (MFPC) learning approach after

**Definition 12**  (MFPC membership function [86,87]). *Let θ∈R be a datum (measurement). Its membership to the fuzzy set, which is determined by a parameter vector $p=\left(\theta_{0},C,B,D\right)$, is computed by the Modified-Fuzzy-Pattern-Classifier membership function as*

(7) $$\mu_{\mathrm{MFPC}}\left(\theta ,p\right)=\left\{\begin{array}{ccc} 2^{-d(\theta ,p_{l})}, & & \theta \leq \theta_{0},\\ 2^{-d(\theta ,p_{r})}, & & \theta >\theta_{0}, \end{array}\right.$$

*where d(θ,p) is a distance measure defined as*

(8) $$d(\theta ,p)=\left(\frac{1}{B}-1\right){\left(\frac{\left|\theta -\theta_{0}\right|}{C}\right)}^{D},$$

Vector $p_{l}=\left(C_{l},B_{l},D_{l}\right)$ contains the parameters for the left-hand part of $\mu_{\mathrm{MFPC}}$ and $p_{r}=\left(C_{r},B_{r},D_{r}\right)$ those of the right-hand part, with the membership function’s properties representing its mode ($\theta_{0}$), class border (C), border membership (B), and slope steepness (D). These are the concatenated elements of the parameter vector $p=\left(\theta_{0}\left|\right|p_{l}\left|\right|p_{r}\right)$.

Based on the available (training) data $\theta ={{}^{N}\theta }_{s}$, the parameters are trained as described in the following. The distance measure d(θ,p) represents the distance of the current datum *θ* to the membership function’s mode $\theta_{0}$. The mode is determined as the median of vector $\theta^{\prime }=\left(\theta^{\prime }\left[1\right],\theta^{\prime }\left[2\right],\cdots ,\theta^{\prime }\left[N\right]\right)$, which is the vector of training data ***θ*** sorted in increasing order, hence: $\theta^{\prime }\left[1\right]\leq \theta^{\prime }\left[2\right]\leq \cdots \leq \theta^{\prime }\left[N\right]$. Then, $\theta_{0}$ is determined by:

(9) $$\theta_{0}=\left\{\begin{array}{ccc} \theta \left[\frac{N+1}{2}\right], & & N\mathrm{odd},\\ \frac{1}{2}\left(\theta \left[\frac{N}{2}\right]+\theta \left[\frac{N}{2}+1\right]\right), & & N\mathrm{even}. \end{array}\right.$$

The border parameters $C_{l/r}$ are determined by:

(10) $$C_{l}=\theta_{0}-\theta_{\min}+{p_{C_{e}}}_{,l}\cdot \left(\theta_{\max}-\theta_{\min}\right),\;C_{r}=\theta_{\max}-\theta_{0}+{p_{C_{e}}}_{,r}\cdot \left(\theta_{\max}-\theta_{\min}\right),$$

where ${p_{C_{e}}}_{,l/r}\in \left[0,1\right]$ are denoted by *percental elementary fuzziness* and represent user-defined width adjustment grades. These are utilised individually to adjust the left- and right-handed function borders, respectively. Thus, $p_{C_{e}}$ provides means to manually change the width of the membership function based on expert knowledge about the respective application after the training process is completed.

Parameter B∈(0,1] determines the membership function’s value on the borders $\theta =\theta_{0}-C_{l}$ and $\theta =\theta_{0}+C_{r}$. For MFPC, this parameter is defined as B=0.5, describing the rising and falling edges of this function by $\mu_{\mathrm{MFPC}}\left(\theta_{0}\pm C,p\right)=B=0.5$. The integer-valued parameter *D* is a user-defined parameter. It is chosen typically as a power of 2 to keep computation of the distance measure $d\left(\theta ,p\right)$ hardware-efficient [86,88]. The exact value is heuristically determined and tuned based on expert knowledge. Low variations in the data require a high value of *D* and vice versa.

**Figure 2:**
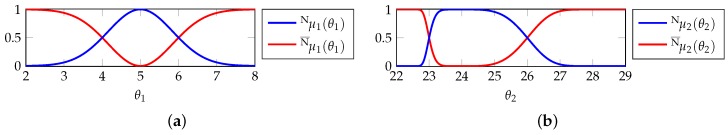
Fuzzy membership functions ${}^{N}\mu_{s}\left(\theta_{s}\right)$ and ${}^{\bar{N}}\mu_{s}\left(\theta_{s}\right)$ for two exemplary sensor measurements representing their normal and abnormal conditions, respectively. (**a**) Normal and abnormal condition for sensor $S_{1}$; (**b**) Normal and abnormal condition for sensor $S_{2}$.

The fuzzy membership function is then applied to determine the grade of membership ${}^{N}\mu_{s}\left(\theta \right)={{}^{N}\mu }_{s}\left(\theta ,p_{s}\right)$, to which a sensor’s measurement *θ* represents the normal condition. The membership function representing abnormal condition ${}^{\bar{N}}\mu_{s}\left(\theta \right)$ follows based on Equation (6). Examples of the membership functions are depicted in Figure 2.

### 3.4. Fuzzified Balanced Two-Layer Conflict Solving

Dempster defined a BBA as belief to a proposition *A*, which is formed by a number of basic elements θ∈A [9]. In analogy to this original definition, a BBA is to be defined for every possible *measurement interval* *A*, in which sensor signals are considered to appear. However, this does not imply the determination of a BBA for a *single measurement value* θ∈A. This is achieved by the solution proposed in [18]. It employs standard fuzzy sets to determine the BBA of θ∈A by its fuzzy membership. This leads to a direct one-to-one relationship between fuzzy memberships ${}^{A}\mu$ and basic belief assignments *m*. The constraints which the membership function necessarily has to satisfy are defined as follows:

**Definition 13** (Constraints on fuzzy basic belief assignment).

1. *The membership function ${}^{A}\mu$ has finite support on the considered frame of discernment* Θ*, i.e., the frame of discernment is finite. Hence, ${}^{A}\mu \left(⊥\right)=0$ and ${}^{A}\mu \left(⊤\right)=0$, where* ⊥ *denotes the smallest element in* Θ*, and* ⊤ *the largest, respectively.*
2. *Fuzzy set A is a standard fuzzy set, i.e., its membership function ${}^{A}\mu$ is unimodal and normal (cf. Definition* 11*). This ensures that its α-cuts form nested sets. Only in this case a transfer between fuzzy memberships to basic belief assignments is possible and valid.*

Then, the process denoted by *fuzzy basic belief assignment* (*μ*BBA) is defined as follows [18]:

**Definition 14** (Fuzzy basic belief assignment (*μ*BBA)). *Let $A_{1},A_{2},A_{3}\in A$ be the α-cuts of fuzzy set A at levels $\alpha_{1}=1$, $\alpha_{2}={}^{A}\mu \left(\theta \right)$, and $\alpha_{3}=\varepsilon >0$ with θ∈Θ and ε≪1, respectively. Since ${}^{A}\mu$ is the fuzzy membership function of the standard fuzzy set A, the α-cuts $A_{1}\subset A_{2}\subset A_{3}$ are the consonant focal elements over A, where $m(A_{i})\neq 0$ for all i. Then the fuzzy basic belief assignment (μBBA) under the constraints of Definition 13 is*

(11) $$\lim_{\varepsilon \to 0}m\left(\theta \right)={}^{A}\mu \left(\theta \right)\;\forall \theta \;\exists {}^{A}\mu \left(\theta \right)\in \left(\varepsilon ,1\right].$$

This BBA determination process is involved in the *fuzzified balanced two-layer conflict solving* (*μ*BalTLCS) fusion operator. It utilises the BalTLCS fusion algorithm to process individual BBAs determined by the *μ*BBA approach.

Consequently, *μ*BalTLCS determines m(θ), i.e., the combined belief in an element θ∈A contained in a proposition, instead of determining the combined belief in a proposition m(A):

**Definition 15** (Fuzzified balanced two-layer conflict solving (*μ*BalTLCS)). *Let A⊆Θ be a proposition from the frame of discernment and θ∈A an element contained in the proposition, and $m_{s}\left(A\right)=m_{s}\left(\theta \right)$ be the BBA, which sensor $S_{s}$ assigns to θ. If $m_{s}\left(\theta \right)$ was determined following the fuzzy basic belief assignment approach and satisfies its constraints (cf. Definition* 14*), then $m_{s}\left(\theta \right)={}^{A}\mu_{s}\left(\theta \right)$. If then a number of such BBAs are fused by BalTLCS after Equation* (4)*, the fusion is defined as a mapping ${}^{A}\mu :{\left[0,1\right]}^{n}\to \left[0,1\right]$ and denoted by fuzzified balanced two-layer conflict solving (μBalTLCS) with*

(12) $${}^{A}\mu ={}^{A}\mu_{\mathrm{nc}}+{}^{A}\mu_{c}.$$

In the following, the notation ${}^{A}\mu_{s}\left(\theta \right)$ is simplified to ${}^{A}\mu_{s}$ for the sake of readability.

It is next shown that *μ*BalTLCS is a fuzzy aggregation operator. Every fuzzy aggregation operator ${}^{A}\mu$ must necessarily satisfy the following three axioms [85]:

**Axiom 3** (Boundary conditions). If ${}^{A}\mu_{s}=0$ for all s, then the aggregated ${}^{A}\mu =0$. Also, if ${}^{A}\mu_{s}=1$ for all s, then the aggregated ${}^{A}\mu =1$.

**Axiom 4** (Increasing Monotonicity). *For any pair $\left({}^{A}\mu_{1},{}^{A}\mu_{2},\cdots ,{}^{A}\mu_{n}\right)$ and $\left({}^{A}\mu_{1}^{\prime },{}^{A}\mu_{2}^{\prime },\cdots ,{}^{A}\mu_{n}^{\prime }\right)$ of n-tuples so that ${}^{A}\mu_{s},{}^{A}\mu_{s}^{\prime }\in \left[0,1\right]$ for all $s\in N_{n}$, and ${}^{A}\mu_{s}\leq {}^{A}\mu_{s}^{\prime }$, then*

$${}^{A}\mu \leq {}^{A}\mu^{\prime }.$$

**Axiom 5** (Continuity). *Let ${}^{A}\mu_{s}\in \left[0,1\right]$. Then ${}^{A}\mu$ is a *continuous* function, if an arbitrary small change ε∈R with 0<ε≪1 of any ${}^{A}\mu_{s}$, i.e., $\left({}^{\varepsilon }\mu_{1},\cdots ,{}^{\varepsilon }\mu_{s},\cdots ,{}^{\varepsilon }\mu_{n}\right)=\left({}^{A}\mu_{1},\cdots ,{}^{A}\mu_{s}+\varepsilon ,\cdots ,{}^{A}\mu_{n}\right)$, results in a small change in ${}^{A}\mu$. That is:*

$$\lim_{\varepsilon \to 0}{}^{\varepsilon }\mu ={}^{A}\mu .$$

These axioms are satisfied by *μ*BalTLCS, which consequently is a fuzzy aggregation operator. The proofs are found in Appendix C.

*μ*BalTLCS further satisfies *symmetry* [85]:

**Axiom 6** (Symmetry). *For any permutation p on $N_{n}$ of $\left({}^{A}\mu_{1},{}^{A}\mu_{2},\cdots ,{}^{A}\mu_{n}\right)$, so that $\left({}^{A}\mu_{1}^{\prime },{}^{A}\mu_{2}^{\prime },\cdots ,{}^{A}\mu_{n}^{\prime }\right)=\left({}^{A}\mu_{p(1)},{}^{A}\mu_{p(2)},\cdots ,{}^{A}\mu_{p(n)}\right)$, holds:*

$${}^{A}\mu ={}^{A}\mu^{\prime }.$$

**Proof.**  The mathematical operations involved in *μ*BalTLCS are addition and multiplication. These are commutative operations, hence the order of the inputs is irrelevant and the result is the same. □

Satisfaction of Axiom 6 is not necessary for an operation to qualify as fuzzy aggregation operator. Nevertheless, it expresses that the respective operator treats the inputs, which are to be aggregated, equally important.

By satisfying the *idempotency* axiom, a fuzzy aggregation operator is called *averaging operator* ([85]:

**Axiom 7.**  *The fuzzy aggregation operator is called idempotent if*

$${}^{A}\mu =\theta$$

*in case of ${}^{A}\mu_{s}=\theta$ with θ∈[0,1] for all s.*

This axiom is not satisfied by *μ*BalTLCS (cf. Equation (C1), where ${}^{A}\mu \neq \theta$). Thus, *μ*BalTLCS is not idempotent and therefore no averaging operator.

It follows that BalTLCS, which is defined in the scope of Dempster-Shafer theory of evidence, is transferred to the framework of fuzzy set theory by utilisation of *μ*BBA. BalTLCS’ applicability is thus increased and not limited to DST-compatible problems. In addition, the conflict determination and handling mechanisms are transferred from Dempster-Shafer theory of evidence and are applicable also in a fuzzy set theory-based setting. These properties are utilised on the attribute layer of MACRO.

### 3.5. MACRO Attribute Layer Fusion

MACRO’s attribute layer fusion approach makes use of the concepts, which are described in Section 3.3 and Section 3.4. This is on the one hand a fuzzy information model to represent the normal condition. This model is parameterised based on measurement data (cf. Definition 12). It is on the other hand the application of the fuzzy *μ*BalTLCS aggregation operation as fusion algorithm on attribute layer. Its inputs are the aforementioned MFPC membership functions, which satisfy the constraints under which *μ*BBA is proposed. Thus, the applicability of *μ*BalTLCS is valid and proposed in this article to determine the attribute health, i.e., an attribute’s grade of membership to the normal condition. The *μ*BalTLCS fusion with respect to attribute *a* is thus given by [20]

(13) $${}_{a}^{N}\mu ={}_{a}^{N}\mu_{\mathrm{nc}}+{}_{a}^{N}\mu_{c},$$

where

(14) $${}_{a}^{N}\mu_{\mathrm{nc}}=\frac{1}{\mathrm{Bc}(n)}\sum_{s=1}^{n-1}\sum_{t=s+1}^{n}{{}^{N}\mu }_{s}\cdot {{}^{N}\mu }_{t},\;{}_{a}^{N}\mu_{c}=c_{a}\cdot \frac{1}{n}\sum_{s=1}^{n}{{}^{N}\mu }_{s},$$

and

(15) $$c_{a}=2\cdot \left(\frac{1}{n}\sum_{s=1}^{n}{{}^{N}\mu }_{s}-{}_{a}^{N}\mu_{\mathrm{nc}}\right).$$

As the abnormal condition is modelled by the normal condition’s complement with ${{}^{\bar{N}}\mu }_{s}=1-{{}^{N}\mu }_{s}$ (cf. Equation (6)), the frame of discernment is assigned no belief in all cases (${{}^{\Theta }\mu }_{s}=0$ for all *s*, cf. Equation (5)) and is thus omitted in the following elaborations. In addition, MACRO’s fusion on attribute layer is carried out by considering only the BBAs assigned to the normal condition due to the mapping ${}^{\bar{N}}\mu_{s}\to {}^{N}\mu_{s}$.

Conflict in a fusion process represents inherent uncertainty. Therefore the information of the applied sensors and consequently the information contained in the result of the attribute’s fusion is not 100% reliable. Conflict is represented by the conflicting coefficient $c_{a}$, an important component of attribute fusion. Thus, the *importance measure* $I_{a}$ of the attribute *a* is defined as follows:

**Definition 16**  (Importance measure [19]). *Let $I_{a}$ be the information weight in a fusion process, which estimates the impact of a conflict regarding the aggregation of sensor information in attribute a. Let ${}_{a}^{N}\mu$ be the fused result of a μBalTLCS process regarding proposition ${}^{N}C$ with the conflicting coefficient $c_{a}\in \left[0,1\right]$. Then $I_{a}:c_{a}\to \left[0,1\right]$ is the corresponding information weight of the fusion result ${}_{a}^{N}\mu$, which is dependent on the attribute’s conflicting coefficient $c_{a}$. The information weight is denoted by importance measure. It is determined by*

(16) $$I_{a}=1-c_{a}.$$

The importance is the complement of the conflicting coefficient. This expresses that the fusion result is more important the less conflict is determined during fusion, and vice versa.

With respect to computational complexity, *μ*BalTLCS is of $O\left(n^{2}\right)$. This has been improved to $O\left(n\right)$ by transferring the original formulation to a matrix formulation and subsequent matrix decomposition. Details on the complexity analysis and improvement are found in [20].

The importance determined for each attribute is—besides each attribute’s health ${}_{a}^{N}\mu$—forwarded to the system layer. A fuzzy fusion algorithm is applied there, which is capable of discounting attributes inheriting large amount of conflict. Details are presented in the next section.

### 3.6. System Layer Fusion

An attribute’s importance $I_{a}\in \left[0,1\right]$ represents the weight of an attribute in the fusion on system layer: the higher an attribute’s importance, the more the attribute influences the system fusion result. The importance of a MACRO attribute is determined continuously based on the conflict between the attribute’s inputs during *μ*BalTLCS fusion (cf. Definition 16). Hence, this information is to be incorporated on system layer during determination of the system’s state. It is noted that manual determination of the importance is also possible, e.g., *a priori* (by an expert) and set statically. A dynamic approach is nevertheless more beneficial as dynamic changes of the monitored system (change of the system’s operation point, varying sensor reliabilities, etc.) are considered.

The employment of importances is considered in the subsequent fusion of the attributes’ healths ${}_{a}^{N}\mu$. These are aggregated on system level using the *implicative importance weighted ordered weighted averaging* (IIWOWA) operator [89] to reason about the entire system under supervision. This operator is based on the *importance weighted ordered weighted averaging* (IWOWA):

**Definition 17**  (Importance weighted ordered weighted averaging [89]). *Let $\mu =(\mu_{1},\mu_{2},\cdots ,\mu_{n})$ be a vector of fuzzy memberships, and $I=(I_{1},I_{2},\cdots ,I_{n})$ a vector of corresponding importance weights. The vector of weights $w=\left(w_{1},w_{2},\cdots ,w_{n}\right)$ determines whether the operator behaves more like the max or more like the min aggregation (more optimistic or more pessimistic), with*

$$\sum_{j=1}^{n}w_{j}=1\;\mathrm{and}\;\rho \left(w\right)=1-\frac{1}{n-1}\sum_{j=1}^{n}\left(n-j\right)\cdot w_{j},$$

*where ρ(w)∈[0,1] determines the aggregation’s andness degree. This is a measure indicating to which degree the operator behaves like the min operation. An andness of ρ(w)=0 represents a pure max, ρ(w)=1 a pure min operation. The operator is able to model any degree of andness between ρ=1 and ρ=0 [90]. Then the class of importance weighted ordered weighted averaging operators is defined as*

(17) $$h_{\mathrm{IWOWA}}\left(I,w,\mu \right)=\sum_{j=1}^{n}w_{j}\cdot b_{\left(j\right)},$$

*with $j\in N_{n}$, and $b_{j}=\rho \left(w\right)+I_{j}\cdot \left(\mu_{j}-\rho \left(w\right)\right)$, where (·) denotes a permutation on b with $b_{\left(1\right)}\geq b_{\left(2\right)}\geq \cdots \geq b_{\left(n\right)}$, i.e., the importance weighted memberships sorted in decreasing order.*

Larsen showed in [89] that the class of IWOWA operators is order-equivalent to the *weighted arithmetic mean* (WAM) operator. Order-equivalence is sufficient when the operator is applied to provide preference ordering [91]. However, in situations where the aggregated value is used for other purposes, such as information fusion, full value-equivalence to WAM is necessary. This property is obtained by normalising Equation (17) in the interval of $h_{\mathrm{IWOWA}}\left(I,w,0\right)$ and $h_{\mathrm{IWOWA}}\left(I,w,1\right)$. This leads to the class of IIWOWA operators:

**Definition 18**  (Implicative importance weighted ordered weighted averaging [91]). *Let 0=(0,⋯,0) be a vector of zeros and 1=(1,⋯,1) a vector of ones, each of length n. Then the class of importance weighted ordered weighted averaging operators is defined with Equation (*17*) as*

(18) $$h_{\mathrm{IIWOWA}}\left(I,w,\mu \right)=\frac{h_{\mathrm{IWOWA}}\left(I,w,\mu \right)-h_{\mathrm{IWOWA}}\left(I,w,0\right)}{h_{\mathrm{IWOWA}}\left(I,w,1\right)-h_{\mathrm{IWOWA}}\left(I,w,0\right)}.$$

In the scope of MACRO, the result of $h_{\mathrm{IIWOWA}}\left(I,w,{}^{N}\mu \right)$ is denoted by *system health*${}^{N}\mu$, where $w={\left(w_{1},w_{2},\cdots ,w_{n}\right)}^{T}\mathrm{with}w_{i}\in \left[0,1\right]$ is a vector of *ordered weighted averaging* (OWA) weights, $I=(I_{1},I_{2},\cdots ,I_{n})$, $I_{a}\in \left[0,1\right]$ a vector of attribute importances, and ${}^{N}\mu =\left({}_{1}^{N}\mu ,{}_{2}^{N}\mu ,\cdots ,{}_{n}^{N}\mu \right)$, ${}_{a}^{N}\mu \in \left[0,1\right]$ a vector of attribute healths. The entire approach facilitates that faulty sensors, which are in contradiction with the other fault-free sensors, do not affect the overall fusion result to a large extent. This is achieved first by the *μ*BalTLCS fusion, which inherently detects and handles conflicts between inputs. In addition, the amount of conflict determined by *μ*BalTLCS is forwarded to the subsequent IIWOWA fusion operation on system layer. Here, attributes full of conflict are devaluated because their conflict is interpreted as uncertainty connected with the attribute. Consequently, attributes containing no or only a small amount of conflict are regarded as important and contribute more to the system health than the unimportant attributes, which are full of conflict. Hence, the confidence of the overall result is increased compared to fusion approaches not incorporating such mechanisms. Although defective sensors immediately influence the conflict/importance, a defective sensor cannot be determined directly within MACRO. For this purpose a solution is proposed in [22].

The *system layer fusion’s degree of optimism* is chosen as follows:

- If the attributes are *significantly dependent* on each other, their information is redundant to a high degree, and external effects affect many or all attributes at the same time. Then the system layer fusion is carried out with a *high degree of optimism*. This leads to a degradation of the system health only when all attributes determine a deterioration of the system state. Consequently, the system health follows the *largest* attribute health.
- If the attributes are *significantly independent* from each other, their information is redundant to a small degree, and external effects affect only some or one attribute. Then the system layer fusion is carried out with a *low degree of optimism*. This leads to a degradation of the system health when at least one attribute determines a deterioration of the system state. Consequently, the system health follows the *smallest* attribute health.

Attribute dependency is only related to the assignment of sensors to the respective attributes. Physical correlations between the sensor signals are irrelevant in this context.

The class of IIWOWA fuzzy aggregation operators is able to model each possible degree of optimism by its andness ρ(w). In accordance to the aforementioned constraints, low andness $\left(\rho (w)\to 0\right)$ is chosen in case of depending attributes, and high andness $\left(\rho (w)\to 1\right)$ in case of independent attributes. Whether any andness degree in between is more appropriate must be decided based on the application. If no information about the degree of dependence is known, an andness of ρ(w)=0.5 resulting in the arithmetic mean is appropriate as initial parameterisation, which may be adjusted later on.

MACRO is completely defined at this point. It is evaluated in the following section.

## 4. Evaluation

The MACRO system is evaluated in the scope of a printing unit demonstrator application for machine condition under laboratory conditions. Its general performance in a real-world scenario with the availability of only a small set of training examples lacking negative examples is shown. In addition, the evaluation illustrates MACRO’s conflict-solving capability. The background of the application is described in the following example.

**Figure 3:**
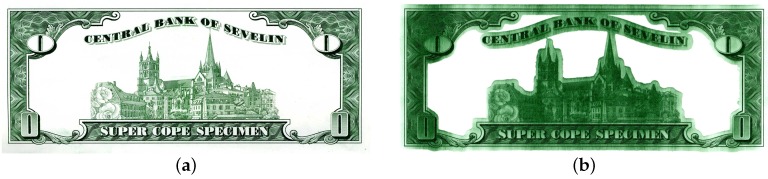
Effect of wiping errors in the intaglio printing process [92]. (**a**) Error-free intaglio print result; (**b**) Print errors caused by wiping errors.

**Example 2** (Printing unit demonstrator). *Intaglio is the major printing process to produce security prints like banknotes. Engraved structures in the printing plates, which are mounted on a rotating plate cylinder, are filled with ink, which is transferred onto the printing substrate under high pressure. A second cylinder denoted by wiping cylinder, which is working in the printing unit, is lubricated with a solvent to wipe off surplus ink from the printing plates by rotating in the direction opposite to the plate cylinder. This process is crucial as wiping errors immediately lead to print errors as shown in Figure 3.*

**Figure 4:**
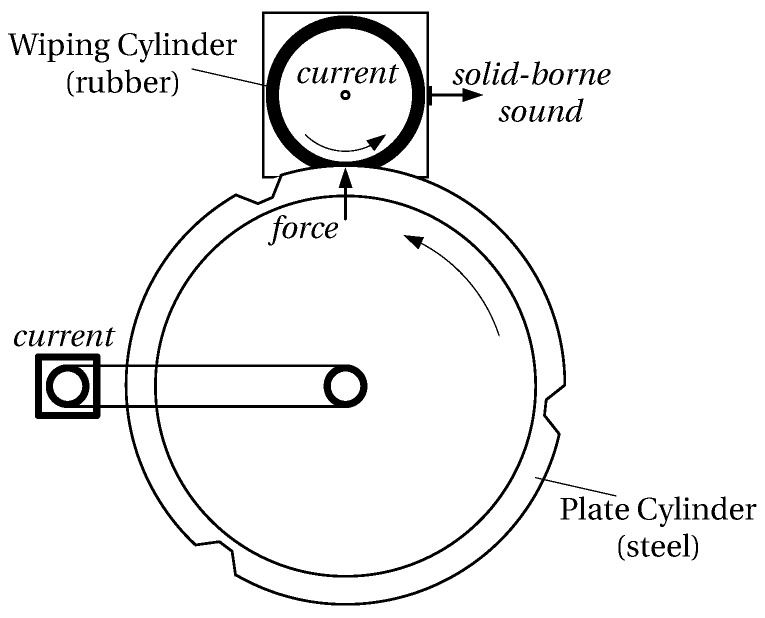
Structural design of the printing unit simulator along with the applied sensors (printed in italic) [92].

The printing unit demonstrator simulates the wiping process. It contains models of the two cylinders, which are turned by electric drives. The pressure between the rubber-surfaced wiping cylinder having a rubber surface and the steel-surfaced plate cylinder is freely adjustable. Four analogue sensors (force, solid-borne sound, electric current of each drive) continuously acquire data during operation to monitor the process. The demonstrator setup is schematically shown in Figure 4.

The printing unit demonstrator is utilised for two experiments. In the first experiment, the demonstrator is operated without any changes, whereas the demonstrator as well as one of the involved sensors is manipulated in the second experiment to enforce a conflict between the sensor signals. The data acquired during the first experiment is assigned to the PU${}_{\mathrm{static}}$ data set and that of the second experiment to the PU${}_{\mathrm{manip}}$ data set. Both sets were evaluated using MACRO, the naïve Bayes, and the Support Vector Machine algorithms in order to deduce the current condition of the demonstrator.

Details on the characteristics of the data, which was acquired during the operation of the demonstrator, are given in Appendix A. Both the raw sensor signal data and the extracted features utilised for evaluation are available online at Zenodo in the data set denoted by “Printing Unit Condition Monitoring” (doi:10.5281/zenodo.55227) [93].

The evaluation setup is detailed in the next section.

### 4.1. Evaluation Setup

The instances in both printing unit demonstrator data sets are not labelled. Nevertheless, the printing unit demonstrator is not manipulated and is considered to be operating in normal condition ${}^{N}C$ at least at the beginning of both experiments: Considering the PU${}_{\mathrm{static}}$ data set, all instances contained in this set represent ${}^{N}C$, whereas in the PU${}_{\mathrm{manip}}$ data set the first manipulation of the printing unit demonstrator begins at k=129 (cf. Appendix A). Therefore, the first 100 instances of each data set are utilised for training. The whole data set is normalised before further processing based on normalisation parameters determined from the training instances. Afterwards, these instances are utilised to train the fuzzy membership functions applied for *μ*BalTLCS fusion in MACRO’s attributes.

It is the task of the evaluated algorithms to assess the instances with respect to its compatibility to the normal condition of the printing unit demonstrator. Changes in the operation condition affecting the actual condition are to be detected.

The MACRO experiment results presented in Section 4.2 and Section 4.3 are obtained with parameter $p_{C_{e}}$ set identically for each membership function to ${p_{C_{e}}}_{l,r}=75\%$. This setting intentionally allows variations of the sensor signals during the demonstrator operation additional to the variations covered during the training phase. The system layer fusion’s andness degree is set in all cases to ρ(w)=0.4. Based on the distribution of each feature’s values, the membership function parameters $D_{l}$ and $D_{r}$ are set empirically to the values shown in Table 3.

MACRO involves three attributes in order to assess the normal condition ${}^{N}C$ of the printing unit demonstrator. Each attribute is composed of features representing a physical property of the printing unit demonstrator:

**Attribute 1 (Motors):**  The *motors* attribute involves the features of the motors’ electrical currents as well as the index of the solid-borne sound frequency with the highest amplitude. It facilitates assessment of the operation of the motors and its attached mechanical parts. Deteriorations or defects of these parts likely lead to changes in the electric currents and/or vibrations emitted by the parts (cf. [46,94,95]).

**Attribute 2 (Contact Pressure):**  This attribute subsumes all features containing information about the pressure between the wiping and plate cylinder. It contains features of the solid-borne sound intensity, the wiping cylinder motor’s electric current, and the contact force sensor.

**Attribute 3 (Motor Currents):**  Here, the features of the motors’ electric currents are evaluated to assess the energy consumption of the system.

These attributes are applied identically in the evaluations of both the PU${}_{\mathrm{static}}$ and PU${}_{\mathrm{manip}}$ data sets. Table 4 summarises the attributes’ compositions.

The MACRO system has been implemented in *MATLAB* according to the formal definitions given in Section 3.5 and Section 3.6. The evaluation has been carried out with MATLAB/Simulink 2016a (9.0.0.341360) 64-bit for Microsoft Windows from The MathWorks, Inc. (The MathWorks, Inc., Natick, MA, USA) [96].

The following fusion algorithms, which originate from machine learning and classification, are utilised as benchmark algorithms. All benchmark evaluations have been carried out within *Waikato Environment for Knowledge Analysis* (WEKA) [97,98] in order to use established implementations of the benchmark fusion algorithms to which this article’s contributions are compared. It offers a number of algorithms for machine learning disciplines like classification, clustering, or feature selection. WEKA 3.8.0 (Machine Learning Group at the University of Waikato, Waikato, New Zealand) was utilised to generate the benchmark results of the condition monitoring experiment. As only training data for the normal condition ${}^{N}C$ is available in the data sets, all benchmark algorithms are evaluated as one-class classifiers:

**Naïve Bayes:**  This is a fusion algorithm originating from probability theory. It determines a conditional probability following Bayes’ theorem. In the context of the condition monitoring experiments evaluated in this section, the conditional probability $P\left({}^{N}C|f\right)={}^{N}P$ is determined. That is, the probability is computed that the feature vector $f={\left(f_{1},f_{2},\cdots ,f_{5}\right)}^{T}$ represents the normal condition ${}^{N}C$ of the printing unit demonstrator. Two variants of the naïve Bayes algorithm are evaluated, which differ in the form of the applied prior distribution:

**nB${}_{\mathrm{Gauss}}$:** The nB${}_{\mathrm{Gauss}}$ variant models the prior distribution as *normal distribution*. It adjusts the distribution’s mean and standard deviation based on the training data.
**nB${}_{\mathrm{kern}}$:** No certain probability distribution is assumed for the prior distribution. It is instead estimated based on the training data by *kernel density estimation* applying Gaussian kernels.
WEKA implements both variants of naïve Bayes in its NaiveBayes classifier. Details on the background of the implementation are found in [99].
  This classifier (and also all other naïve Bayes implementations found by the authors) is only capable to be applied if data for more than one class is available in the training data. The printing unit demonstrator experiments deliver only data about the demonstrator’s condition, which is *per se* assumed to represent its normal condition. Thus, the naïve Bayes implementation is applied in combination with the WEKA package OneClassClassifier (WEKA packages are conveniently installed by utilisation of its integrated package manager). This is a meta-classifier, which allows to apply any classifier on one-class problems like the printing unit demonstrator condition monitoring experiments: Based on the training data, artificial data representing its counter-class is generated, facilitating to handle the original one-class problem as two-class problem. The result is obtained by the combination of the prior information derived from the training data with the employed classifier’s output. It utilises Bayes’ theorem for this task. For details on the background of OneClassClassifier see [100].

**Support Vector Machine:**  The *Support Vector Machine* (SVM) is a classification concept, which linearly separates the data in an *n*-dimensional hyperspace. Its binary output $g\left(f\right)\in \left\{{}^{N}C,{}^{\bar{N}}C\right\}$ describes whether the feature vector $f={\left(f_{1},f_{2},\cdots ,f_{5}\right)}^{T}$ represents the normal condition ${}^{N}C$ of the printing unit demonstrator. The linear hyperplane is determined based on the training data and encoded in the SVM’s support vectors. It involves kernel functions, which transform the input data into a higher-dimensional space, in which linear separation is possible. In the scope of the printing unit demonstrator experiments, it is applied with a Gaussian *radial basis function* (RBF). This is a parameterisable kernel, whose parameter γ∈R adjusts the kernel’s variance. Details on SVMs is found in [101]. For the printing unit demonstrator condition monitoring experiment, WEKA’s LibSVM package is utilised. It is a wrapper classifier providing access to the libSVM implementation, a free SVM library, which contains a *one-class SVM* implementation, by Chang and Lin [102]. Thus, it applicable to the printing unit demonstrator experiments without further adjustments.

All instances of the printing unit demonstrator data sets are evaluated in the following, i.e., the training data is also evaluated. The next section presents the evaluation results on the PU${}_{\mathrm{static}}$ data set, both obtained by applying MACRO fusion and the benchmark fusion algorithms. Afterwards, the results on the PU${}_{\mathrm{manip}}$ data set are presented in Section 4.3.

### 4.2. PU_static_ Data Set Results

**Figure B1:**
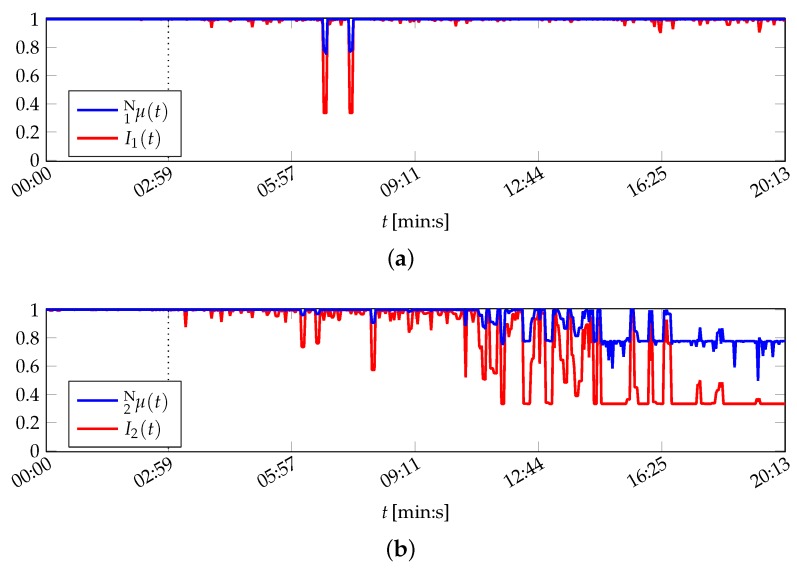

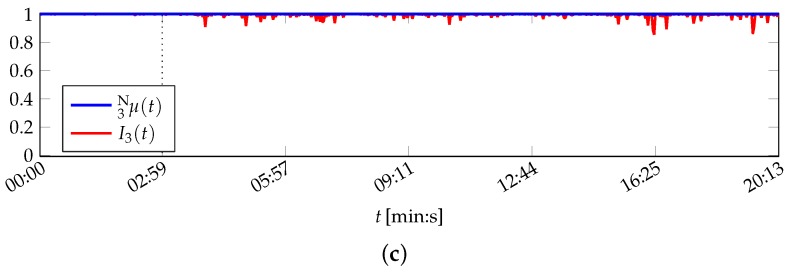
Attribute health evaluation over time during normal operation of the printing unit demonstrator (PU${}_{\mathrm{static}}$). No manipulation or fault occurred during the 20:13 (min:s) operation time. Plots (**a**–**c**) show attribute healths ${}_{a}^{N}\mu \left(t\right)$ and their corresponding importances $I_{a}\left(t\right)$. Variations in the curves are due to effects of the operation itself. Training data was acquired up to 2:59 (min:s) (100 plate cylinder revolutions, cf. the black dotted line). Attribute composition according to Table 4. (**a**) Attribute 1: Motors; (**b**) Attribute 2: Contact Pressure; (**c**) Attribute 3: Motor Currents.

The inputs of the *μ*BalTLCS fusion on MACRO’s attribute layer are the fuzzy memberships of the features, which are extracted from the sensor signals. Plots of the attribute healths are depicted in Figure B1. The three attributes defined in Table 4 are fused by the IIWOWA operator on system layer to obtain the system health ${}^{N}\mu$ of the printing unit demonstrator. Then, all information to assess the current state of the printing unit demonstrator is available from the continuously evolving system health. In order to obtain a crisp decision about the system condition, the system health function ${}^{N}\mu$ is evaluated with respect to the following thresholds ${}^{N}\eta_{i}\in R$:

${}^{N}\mu \geq {}^{N}\eta_{\mathrm{warn}}<1$: In this range, the system operation is considered to be *normal*. Deviations from ${}^{N}\mu =100$% are intentionally allowed as the behaviour of physical systems is usually not constant (e.g., due to variations in the system’s environment).
${}^{N}\eta_{\mathrm{emerg}}\leq {}^{N}\mu <{}^{N}\eta_{\mathrm{warn}}$: If the system health determined during operation is in this range, it is neither considered normal nor in an emergency condition. Instead, it is in a *warning* condition. This state may, for example, be utilised to increase attention of maintenance personnel. This range is considered as a transient area, in which it is likely that a system defect will follow in the future.
${}^{N}\mu <{}^{N}\eta_{\mathrm{emerg}}$: In this case, the system is considered to be in an *emergency* condition. It might already bear a *defect* and appropriate measures, like an emergency stop of the system, have to be taken.

**Figure 5:**
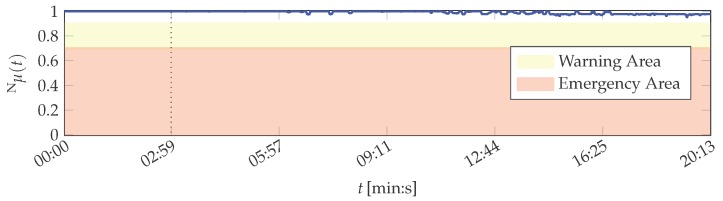
Evaluations of the system health ${}^{N}\mu$ over time during static operation of the printing unit demonstrator (PU${}_{\mathrm{static}}$). The result depicted here is based on the attribute healths shown in Figure B1.

The thresholds are set dependent on the respective application. In the scope of the printing unit demonstrator, these are configured as ${}^{N}\eta_{\mathrm{warn}}=0.9$ and ${}^{N}\eta_{\mathrm{emerg}}=0.7$. The results of the system health including the warning and emergency areas are depicted in Figure 5.

The system health values ${}^{N}\mu$ are greater than 0.94 for all *t*. Hence, the system state is correctly classified as normal for all data set instances, despite of feature variations due to the demonstrator being in its start-up phase: It is visible that both the outliers of attribute 1 and the devaluation of attribute 2, which does not correspond to the real demonstrator condition (cf. Figure B1), have a decreased influence on the result on system layer due to their decreased importance. A slight decrease in system health caused by these effects is nevertheless perceptible, but not to the same amount as they influenced the attribute healths.

**Figure 6:**
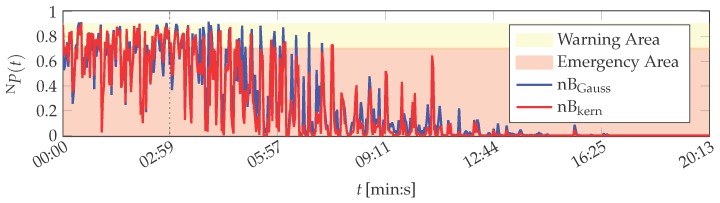
System health evaluation over time during static operation of the printing unit demonstrator (PU${}_{\mathrm{static}}$) by one-class naïve Bayes applying Gaussian (nB${}_{\mathrm{Gauss}}$) and kernel-density estimated (nB${}_{\mathrm{kern}}$) priors.

The features of the PU${}_{\mathrm{static}}$ data set are also evaluated by the probabilistic naïve Bayes algorithm and the SVM. Results obtained by naïve Bayes are depicted in Figure 6.

The variants nB${}_{\mathrm{Gauss}}$ and nB${}_{\mathrm{kern}}$ perform similar. With respect to the training phase (up to 02:59 (min:s)), both variants assess the printing unit demonstrator to be mostly in warning or emergency condition (the same thresholds ${}^{N}\eta$ as in the MACRO evaluation are utilised for the naïve Bayes evaluation). Only 3 of the 100 training instances by nB${}_{\mathrm{Gauss}}$ and 2 by nB${}_{\mathrm{kern}}$ are assessed as normal system operation. After training finished, the naïve Bayes classifiers assess all following instances to be no normal operation. The system health determined by nB${}_{\mathrm{kern}}$ decays in average up to 16:31 min, when it reaches ${}^{N}P=0$ for the remainder of the experiment. The nB${}_{\mathrm{Gauss}}$ variant is more optimistic and yields non-zero probabilities up to 20:05 (min:s).

Altogether, both naïve Bayes approaches do not represent the actual normal operation condition of the printing unit demonstrator. However, they indicate a continuous drift in the system behaviour, which is plausible as the demonstrator is in its start-up phase (cf. Appendix A).

**Figure 7:**
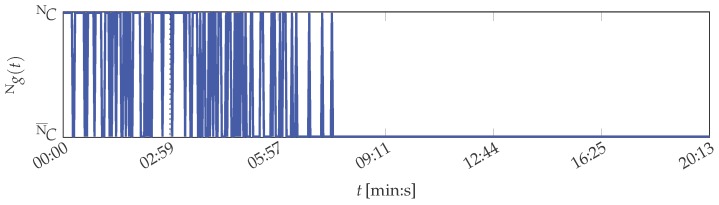
System health evaluation over time during static operation of the printing unit demonstrator (PU${}_{\mathrm{static}}$) by one-class SVM.

The same is concluded for the SVM. Its Gaussian radial basis function kernel is parameterised with $\gamma =5\times 10^{-7}$. With this parameterisation, the SVM achieved minimal classification error for 10-fold cross-validation of the training data. Its evaluation results are depicted in Figure 7.

During the training phase, 26 of the 100 instances are classified as abnormal condition. It further fluctuates between normal and abnormal condition, also after the training phase, without a physical cause. Stable classification of the printing unit demonstrator condition is hence not possible.

The next section evaluates the data collected during the manipulated printing unit demonstrator operation.

### 4.3. PU_manip_ Data Set Results

In contrast with the PU${}_{\mathrm{static}}$ data set, PU${}_{\mathrm{manip}}$ contains data acquired during printing unit demonstrator operation, which also includes a number of intended and unintended influences, which the demonstrator is exposed to (cf. Table A3). In addition, the printing unit demonstrator had already been running for around 23:00 (min:s) before data acquisition started. Thus, the data is assumed to be acquired during operation in a stable operation point of the printing unit demonstrator. With respect to the actual condition of the printing unit demonstrator, this situation is prevailing during the first seven hours and eight minutes of the experiment: During this time no changes in the system’s operation occurred or were induced.

However, filtering of the solid-borne sound sensor started at 03:45 (min:s) by activating an analogue low-pass filter to simulate a sensor defect. Between 03:55 (min:s) and 04:33 (min:s) the cutoff frequency of the filter is continuously decreased until the smallest possible is set. The filter setting is kept until 06:36 (min:s), when the filter is deactivated again. Consequently, the printing unit demonstrator was operated from 06:36 (min:s) on under the same constraints as at the beginning of the experiment. It changed from 07:08 (min:s) on, when uneven rotations of the cylinders were perceptible. This change in the behaviour of the system was not intended and is interpreted as a temporary defect of the demonstrator. The experiment ends by intentionally lifting the wiping cylinder at 08:33 (min:s) in order to cancel contact pressure between the cylinders. This represents a new operation point and not a malfunction, as no defect resulted in the decreased contact pressure.

**Figure 8:**
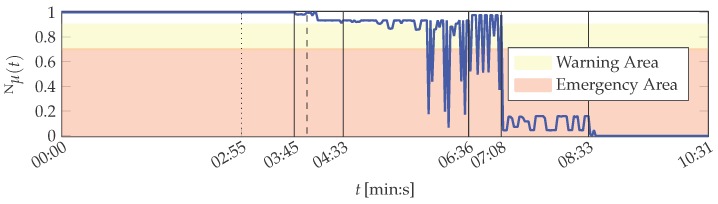
Evaluation of the system health ${}^{N}\mu$ over time during manipulated operation of the printing unit demonstrator (PU${}_{\mathrm{manip}}$). The result depicted here is based on the attribute healths shown in Figure B2.

**Figure B2:**
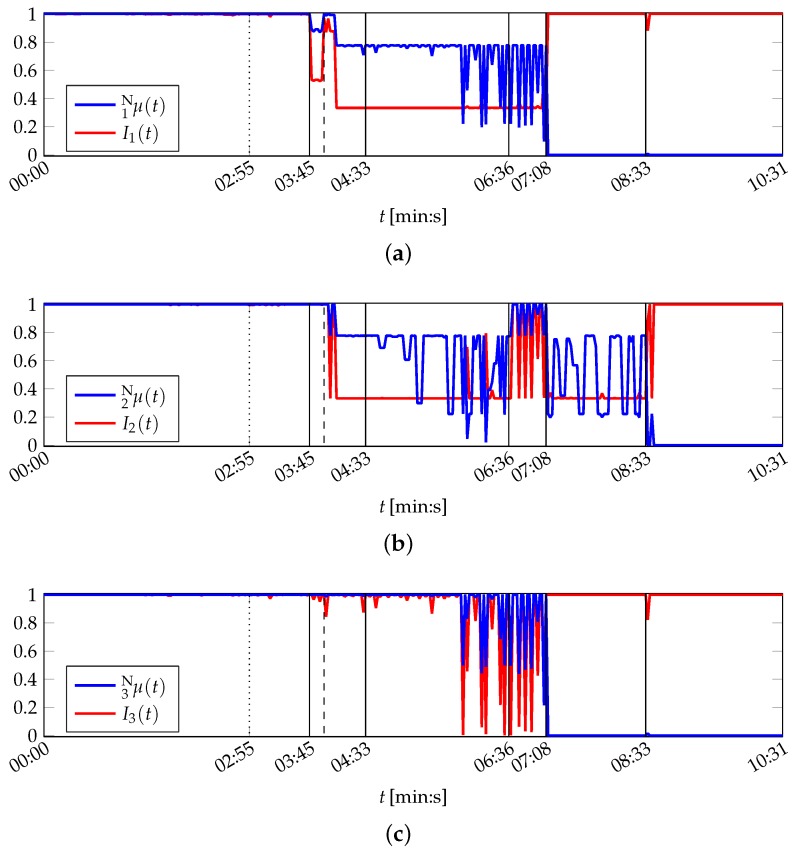
Attribute health evaluation over time during manipulated operation of the printing unit demonstrator (PU${}_{\mathrm{manip}}$). Plots (**a**–**c**) show attribute healths ${}_{a}^{N}\mu \left(t\right)$ and their corresponding importances $I_{a}\left(t\right)$. Training data was acquired up to 2:55 (min:s) (100 plate cylinder revolutions, cf. the black dotted line). Variations in the curves are due to effects of the operation itself and result from manipulations or faults, marked by vertical black lines (cf. Table A3). Attribute composition according to Table 4. (**a**) Attribute 1: Motors; (**b**) Attribute 2: Contact Pressure; (**c**) Attribute 3: Motor Currents.

It is shown in the following that the MACRO system is capable to represent the actual situation of the printing unit demonstrator in its outputs. The MACRO attribute healths of this case are depicted in Figure B2. The resulting system health is depicted in Figure 8. Here, the same warning and emergency thresholds are applied as for PU${}_{\mathrm{static}}$.

The system health is constantly ${}^{N}\mu =1.0$ from the beginning of the experiment until the activation of the low-pass filter. Then ${}^{N}\mu$ starts to decrease, but remains above the warning level ${}^{N}\eta_{\mathrm{warn}}=0.9$ until the minimal cutoff frequency is set at 04:33 (min:s). Hence, the evolving defect of one sensor is compensated by MACRO and does not result in assessing the printing unit demonstrator to be in emergency condition.

In the following period up to 06:36 (min:s) the system health falls into the warning area twice before the system health temporarily also falls in the emergency area. The simulated sensor defect is not the main reason for the decrease of ${}^{N}\mu$. Instead, actual imperceptible variations in the demonstrator’s behaviour affect the motor currents. The variations continue on the one hand between 06:36 (min:s) and 07:08 (min:s), when the printing unit demonstrator’s operation setup is reset by “repairing” the solid-borne sound sensor. On the other hand the average system health value increases again, reflecting the improvement in the sensor equipment.

The uneven rotations of the cylinders between 07:08 (min:s) and 08:33 (min:s) result in low system health values. Its magnitude is limited by attributes 1 and 3, which both are zeroed, whereas the course of ${}^{N}\mu$ follows attribute 2 (cf. Figure B2). Nevertheless, the latter attribute affects the system health only to a small extent due to its decreased importance during this period.

During the remaining time of the experiment, the system health is zeroed, according to the attributes and the printing unit demonstrator’s actual condition: It is completely different from the condition during the training phase due to lifting the wiping cylinder.

**Figure 9:**
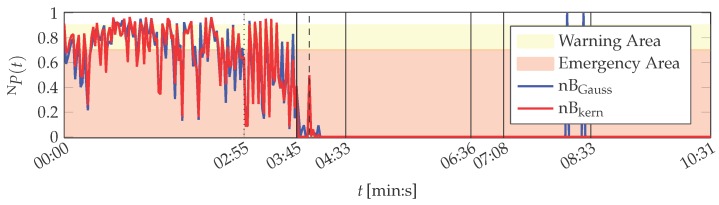
System health evaluation over time during manipulated operation of the printing unit demonstrator (PU${}_{\mathrm{manip}}$) by one-class naïve Bayes applying Gaussian (nB${}_{\mathrm{Gauss}}$) and kernel-density estimated (nB${}_{\mathrm{kern}}$) priors.

The results of the naïve Bayes classification algorithms with respect to the features contained in the PU${}_{\mathrm{manip}}$ data set are visualised in Figure 9.

The printing unit demonstrator is physically in normal condition up to 07:08 (min:s). This is only partly represented in the naïve Bayes classifications before sound filter activation at 03:45 (min:s). During this time, the probabilities ${}^{N}P$ of both variants nB${}_{\mathrm{Gauss}}$ and nB${}_{\mathrm{kern}}$ vary and determine the demonstrator’s condition to be mostly in a warn or emergency state. Only 23 (nB${}_{\mathrm{Gauss}}$) and 25 (nB${}_{\mathrm{kern}}$) data set instances are assigned normal condition.

Along with the activation of the sound filter, the probabilities decrease further. The gradually increasing attenuation of the solid-borne sound signal is also represented in the outputs of the naïve Bayes algorithms: Their probabilities approach ${}^{N}P=0$ in the respective time frame between 03:55 (min:s) and 04:33 (min:s). The probabilities of nB${}_{\mathrm{kern}}$ remain on this level until the end of the experiment, whereas nB${}_{\mathrm{Gauss}}$ classifies two instances representing normal condition (t∈{08:11,08:27} (min:s)). These are assumed to be numerical errors caused by the classifier’s implementation rather than caused by the demonstrator: the plate cylinder turns unevenly during this time and hence the printing unit demonstrator is not in normal operation condition. However, it was not possible to verify this assumption.

Altogether, both naïve Bayes approaches do not represent the actual normal operation condition of the printing unit demonstrator. They are also misled by the simulated solid-borne sound sensor defect, which does not affect the true physical condition of the printing unit demonstrator.

**Figure 10:**
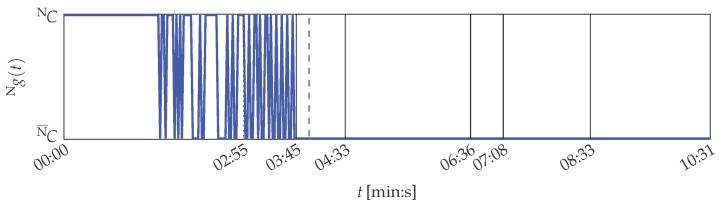
System health evaluation over time during manipulated operation of the printing unit demonstrator (PU${}_{\mathrm{manip}}$) by one-class SVM.

Similar results are obtained during SVM evaluation. The SVM’s Gaussian radial basis function kernel is parameterised with $\gamma =5\times 10^{-7}$. With this parameterisation, the SVM achieved minimal classification error for 10-fold cross-validation of the training data. Its evaluation results are depicted in Figure 10.

The SVM constantly classifies the instances in the training data to represent normal condition up to 01:31 (min:s). Then, the SVM begins to vary in its decision, until its decision is ${}^{\bar{N}}C$ from 03:45 (min:s) on. It is thus correct in its decisions from 07:08 (min:s) onwards, when the printing unit demonstrator is actually no more in the condition in which it was during the training phase. Nevertheless, the SVM approach is unstable shortly after the beginning of the experiment in its decisions. It is also misled by the solid-borne sound sensor defect.

The results obtained during the printing unit demonstrator condition monitoring experiments are discussed in the following section.

## 5. Discussion of the Results

The experiments in the scope of the printing unit demonstrator show the benefits of MACRO fusion for condition monitoring compared to naïve Bayes and an SVM. Whereas the outputs of the naïve Bayes and SVM algorithms fluctuate even for the training data, the MACRO output is nearly constant during the training phase and is in general more stable. MACRO is further able to compensate the simulated solid-borne sound sensor defect: in contrast to naïve Bayes and SVM, the sensor defect does not lead to a decision that the observed printing unit demonstrator condition is not normal. Hence, the fusion results of MACRO in the scope of the printing unit demonstrator experiments represent the true physical condition best.

## 6. Conclusions

The handling of conflicts between information sources is crucial for the reliability of the result of an information fusion application. This article presents the two-layer *multilayer attribute-based conflict-reducing observation* (MACRO) information fusion system. The article focuses on its attribute layer fusion algorithm *fuzzified balanced two-layer conflict solving* (*μ*BalTLCS), which is capable to determine conflicts between fusion inputs and decrease their effect on the fusion result. It originates from Dempster-Shafer theory of evidence and operates on fuzzy sets, which represent an attribute’s normal condition. The validity of *μ*BalTLCS’s usage in a fuzzy set theory context is proved. Its conflict-solving capability is validated in the evaluation of a condition monitoring application. It is shown that MACRO represents the true physical condition, whereas naïve Bayes and SVM fusion yield less stable results and are misled by the sensor manipulation: Although this simulated defect does not affect the physical condition of the printing unit demonstrator, it is classified to be in abnormal condition.

The BalTLCS fusion operation is a generally applicable fusion operator, which offers intuitive results in high-conflict situations, in the scope of DST. All information fusion problems, which are expressible in terms of BBAs, are suitable to be handled with this. In addtion, its fuzzified variant *μ*BalTLCS can be applied in any fuzzy aggregation application where conflicts need to be considered. Fields of application for MACRO cover all use cases, where a condition is to be monitored. These use cases include human health monitoring, smart grid supervision, or on-chip state monitoring on SoCs, for example. Monitoring of more than one state is also possible by the set-up of one MACRO instance per state.

Learning and maintenance of the model for the normal condition is an open question for future research as the procedure to update the parameterisation in order to adapt to the current situation is not yet defined. Algorithm optimisations, which bring the *μ*BalTLCS algorithm one step closer to support implementations on suitable hardware platforms, are presented in [20]. Along with its matrix-based regular structure, a formulation, which is beneficial for close-to-hardware implementations on embedded devices, is provided. In addition, MACRO and *μ*BalTLCS bear potential for parallelisation, which further supports real-world embedded device implementations. However, an actual hardware implementation has not yet been achieved and is part of future considerations. A major open topic in the context of MACRO is the automatic design, update, and adaptation of the information fusion process. Preliminary research on this has been conducted and published [23,24], but does not completely cover the important topic.

## Figures and Tables

**Table 1:** Physicians’ beliefs about a patient’s disease (according to [65]).

*Disease*	Meningitis	Brain Tumor	Concussion
*Doctor A*	0.99	0.01	0.00
*Doctor B*	0.00	0.01	0.99

**Table 2:** Fusion result of Dempster’s rule of combination (DRC) given the individual beliefs presented in  Table 1.

*Disease*	Meningitis	Brain Tumor	Concussion
*DRC*	0.00	1.00	0.00

**Table 3:** Membership function parameters of the features with respect to the printing unit demonstrator condition monitoring data sets.

	RD${}_{\mathrm{static}}$			RD${}_{\mathrm{manip}}$	
**Feature**	$D_{l}$	$D_{r}$		$D_{l}$	$D_{r}$
$f_{1}$: arithmetic mean of the contact force	16	8		8	20
$f_{2}$: root mean square of the solid-borne sound (sound intensity)	16	8		20	8
$f_{3}$: index of the frequency component with largest amplitude	16	16		8	16
$f_{4}$: arithmetic mean of the wiping cylinder motor current	16	16		16	8
$f_{5}$: arithmetic mean of the plate cylinder motor current	8	16		16	8

**Table 4:** MACRO attribute compositions for the printing unit demonstrator experiment.

Attribute	Attribute Description	Number of Features	Features
1	motors	3	$f_{3}$, $f_{4}$, $f_{5}$
2	contact pressure	3	$f_{1}$, $f_{2}$, $f_{4}$
3	motor currents	2	$f_{4}$, $f_{5}$

## Supplementary Materials

The data set utilised for evaluations is available online: https://zenodo.org/record/55227 at Zenodo denoted by “Printing Unit Condition Monitoring” (doi:10.5281/zenodo.55227) [93].

## Abbreviations

The following abbreviations are used in this article:

*μ*BBA	fuzzy basic belief assignment
*μ*BalTLCS	fuzzified balanced two-layer conflict solving
AAL	ambient assisted living
BalGCR	balanced group conflict redistribution
BalTLCS	balanced two-layer conflict solving
BBA	basic belief assignment
CMDST	Conflict-Modified-DST
DRC	Dempster’s rule of combination
DST	Dempster-Shafer theory of evidence
FFT	fast Fourier transform
FST	fuzzy set theory
GCR	Group-Conflict-Redistribution
IFU	information fusion
IIWOWA	implicative importance weighted ordered weighted averaging
IWOWA	importance weighted ordered weighted averaging
MACRO	multilayer attribute-based conflict-reducing observation
MFPC	Modified-Fuzzy-Pattern-Classifier
OWA	ordered weighted averaging
pdf	probability density function
PosT	possibility theory
ProbT	probability theory
RMS	root mean square
RTE	redundant target effect
SoC	system on chip
SVM	Support Vector Machine
TLCS	Two-Layer Conflict Solving
WAM	weighted arithmetic mean
WEKA	Waikato Environment for Knowledge Analysis

## Appendix A. Evaluation Data Set Characteristics

**Printing Unit Demonstrator Condition Monitoring:** The behaviour of the printing unit demonstrator during operation is observed by four analogue sensors. They each output a continuous voltage signal in the range of [-10,10] V, which is proportional to the respective quantity the sensor is observing. Thus, each signal’s unit is irrelevant and abandoned as changes of the original quantity of interest are reflected also in the respective voltage signal. All output time-domain signals are synchronously and equidistantly sampled at a frequency of 20 kHz and quantised with a resolution of 16 bit. The acquired data is then split into non-overlapping batches of 50,000 samples (corresponding to 2.5 s of operation), respectively. The length of the time frame was chosen to ensure that 3 revolutions of the plate cylinder are captured in each signal data batch. Each plate cylinder revolution is represented by a data set instance. One of the signals (solid-borne sound) is treated by the *fast Fourier transform* (FFT) to determine its frequency spectrum per signal batch. Altogether, 5 time- and frequency domain signals are available, from which 5 features per plate cylinder revolution are extracted. That is, every instance in the data set is described by a vector of 5 feature values. This results in 15 feature values per signal data batch. Table A1 and Table A2 summarise which signals were acquired and which features were extracted during the printing unit demonstrator operation.

**Table A1:** Summary of the signals *d* acquired during printing unit demonstrator operation.

Symbol	Signal Name	Signal Domain
$d_{1}$	contact force	time
$d_{2}$	solid-borne sound	time
$d_{3}$	solid-borne sound spectrum	frequency
$d_{4}$	motor current wiping cylinder	time
$d_{5}$	motor current plate cylinder	time

**Table A2:** Summary of the features extracted from the sensor signals *d* acquired at the printing unit demonstrator.

Symbol	Feature Name	Feature Description
$f_{1}$	$\mathrm{mean}(d_{1})$	arithmetic mean of the contact force
$f_{2}$	$\mathrm{rms}(d_{2})$	root mean square of the solid-borne sound (sound intensity)
$f_{3}$	$\mathrm{maxAmplFreqInd}(d_{3})$	index of the frequency component with largest power
$f_{4}$	$\mathrm{mean}(d_{4})$	arithmetic mean of the wiping cylinder motor current
$f_{5}$	$\mathrm{mean}(d_{5})$	arithmetic mean of the plate cylinder motor current

The printing unit demonstrator condition monitoring use case is divided into two experiments under different operation conditions:

- **Static printing unit demonstrator operation (PU${}_{\mathrm{static}}$):** The static experiment observes the printing unit demonstrator during 20:13 (min:s) of operation. The printing unit demonstrator was started immediately before the data acquisition began. No additional manipulations or events occurred during the experiment. Therefore, only data representing the demonstrator’s normal condition ${}^{N}C$ is contained in the PU${}_{\mathrm{static}}$ data set. It contains 10,000,000 raw signal samples resulting in 600 instances (plate cylinder revolutions), which are in summary described by 3000 feature values.
- **Manipulated printing unit demonstrator operation (PU${}_{\mathrm{manip}}$):** The printing unit demonstrator was started ca. 23:00 (min:s) before the data acquisition began. During this 10:31 (min:s) long experiment, the demonstrator application was intentionally manipulated. In addition, the solid-borne sound sensor signal was manipulated through low-pass filtering in order to simulate a defect of this sensor. An unintended incident also occurred during this experiment. Therefore, data representing both the demonstrator’s normal and abnormals conditions ${}^{N}C$ and ${}^{\bar{N}}C$ are contained in the PU${}_{\mathrm{manip}}$ data set. The sequence of events along with an objective classification of the demonstrator condition by the human experimenter is summarised in Table A3. The PU${}_{\mathrm{manip}}$ data set contains 5,950,000 raw signal samples, which are in summary described by 1785 feature values.

**Table A3:** Description of the printing unit demonstrator operation and the events, which occurred during the manipulated printing unit demonstrator operation experiment. These are covered by the PU${}_{\mathrm{manip}}$ data set. The demonstrator condition reflects the objective assessment of the printing unit demonstrator by the human experimenter during operation.

Time [min:s]	Instance *k*	Event and Operation Description	Demonstrator Condition
00:00–02:55	1–100	training data acquisition	${}^{N}C$
02:55–03:45	101–128	normal operation without incidents or manipulations	${}^{N}C$
03:45–03:55	129–135	activation of analogue low-pass signal filter to treat the solid-borne sound signal	${}^{N}C$
03:55–04:33	136–155	gradual attenuation of the solid-borne sound signal by continuously decreasing the low-pass filter’s cutoff frequency	${}^{N}C$
04:33–06:36	156–224	normal operation at smallest possible cutoff frequency of the solid-borne sound low-pass filter	${}^{N}C$
06:36–07:08	225–242	deactivation of the analogue low-pass filter	${}^{N}C$
07:08–08:33	243–290	uneven turning of the plate cylinder (unintended)	${}^{\bar{N}}C$
08:33–10:31	291–357	contact pressure decreased until no contact between both cylinders	${}^{\bar{N}}C$

## Appendix B. MACRO Attribute Evaluation Results

This section contains the attribute healths obtained during MACRO evaluation. Figure B1 shows the results from the PU${}_{\mathrm{static}}$ data set, those from the PU${}_{\mathrm{manip}}$ data set are depicted in Figure B2.

## Appendix C. Proofs

**Proof of Lemma 1.**

$$\sum_{i=1}^{o}m\left(A_{i}\right)=\sum_{i=1}^{o}\left(m_{\mathrm{nc}}\left(A_{i}\right)+m_{c}\left(A_{i}\right)\right)=\frac{1}{\mathrm{Bc}(n)}\sum_{s=1}^{n-1}\sum_{t=s+1}^{n}\sum_{i=1}^{o}m_{s}\left(A_{i}\right)\cdot m_{t}\left(A_{i}\right)+c\cdot \frac{1}{n}\sum_{s=1}^{n}\sum_{i=1}^{o}m_{s}\left(A_{i}\right).$$

With $\sum_{s=1}^{n}\sum_{i=1}^{o}m_{s}\left(A_{i}\right)=n$ follows:

$$\begin{array}{cc} \sum_{i=1}^{o}m\left(A_{i}\right) & =\frac{1}{\mathrm{Bc}(n)}\sum_{s=1}^{n-1}\sum_{t=s+1}^{n}\sum_{i=1}^{o}m_{s}\left(A_{i}\right)\cdot m_{t}\left(A_{i}\right)+\frac{1}{\mathrm{Bc}(n)}\sum_{s=1}^{n-1}\sum_{t=s+1}^{n}\sum_{i=1}^{o}m_{s}\left(A_{i}\right)\cdot \left(1-m_{t}\left(A_{i}\right)\right)\\ & =\frac{1}{\mathrm{Bc}(n)}\sum_{s=1}^{n-1}\sum_{t=s+1}^{n}\sum_{i=1}^{o}\left(m_{s}\left(A_{i}\right)\cdot m_{t}\left(A_{i}\right)+m_{s}\left(A_{i}\right)\cdot \left(1-m_{t}\left(A_{i}\right)\right)\right)\\ & =\frac{1}{\mathrm{Bc}(n)}\sum_{s=1}^{n-1}\sum_{t=s+1}^{n}\underset{1}{\underset{︸}{\sum_{i=1}^{o}m_{s}\left(A_{i}\right)}}\cdot \underset{1}{\underset{︸}{\left(m_{t}\left(A_{i}\right)+1-m_{t}\left(A_{i}\right)\right)}}\\ & =\frac{1}{\mathrm{Bc}(n)}\sum_{s=1}^{n-1}\sum_{t=s+1}^{n}1=\frac{1}{\mathrm{Bc}(n)}\sum_{s=1}^{n-1}\left(\sum_{t=1}^{n}1-\sum_{t=1}^{s}1\right)=\frac{1}{\frac{n}{2}\left(n-1\right)}\sum_{s=1}^{n-1}\left(n-s\right). \end{array}$$

With the sum of the arithmetic progression $\sum_{s=1}^{n}s=\frac{1}{2}n\left(n+1\right)\Rightarrow \sum_{s=1}^{n-1}s=\frac{1}{2}\left(n-1\right)n$ (cf. [103] p. 79),

$$\sum_{i=1}^{o}m\left(A_{i}\right)=\frac{1}{\frac{n}{2}\left(n-1\right)}\cdot \frac{n}{2}\left(n-1\right)=1.$$

□

**Proof of Axiom 3**. First ${}^{A}\mu_{s}=0$ for all *s* is considered, i.e., no belief is assigned to proposition *A* by any sensor. As no information is provided about further propositions, the remaining belief is assigned to the frame of discernment to satisfy Definition 5, i.e., ${}^{\Theta }\mu_{s}=1-{}^{A}\mu_{s}=1$ for all *s*. Then

$${}^{A}\mu_{\mathrm{nc}}=\frac{1}{\mathrm{Bc}(n)}\sum_{s=1}^{n-1}\sum_{t=s+1}^{n}{}^{A}\mu_{s}\cdot {}^{A}\mu_{t}=0,\;{}^{\Theta }\mu_{\mathrm{nc}}=\frac{1}{\mathrm{Bc}(n)}\sum_{s=1}^{n-1}\sum_{t=s+1}^{n}{}^{\Theta }\mu_{s}\cdot {}^{\Theta }\mu_{t}=1,$$

$$c=1-\sum_{i=1}^{o}{}^{A_{i}}\mu_{\mathrm{nc}}=1-\left({}^{A}\mu_{\mathrm{nc}}+{}^{\Theta }\mu_{\mathrm{nc}}\right)=0,\;{}^{A}\mu_{c}=c\cdot \frac{1}{n}\sum_{s=1}^{n}{}^{A}\mu_{s}=0.$$

Consequently, ${}^{A}\mu ={}^{A}\mu_{\mathrm{nc}}+{}^{A}\mu_{c}=0$. By evaluation of ${}^{\Theta }\mu$, where ${}^{\Theta }\mu_{s}=1$ for all *s*, the proof is concluded:

$${}^{\Theta }\mu ={}^{\Theta }\mu_{\mathrm{nc}}+{}^{\Theta }\mu_{c}=1.$$

□

**Proof of Axiom 4**. Let ${}^{A}\mu_{s}=\theta$ for all $s\in N_{n}$ and ${}^{A}\mu_{s}^{\prime }=\theta +\varepsilon \leq 1$ for all *s* without loss of generality, where θ∈[0,1] and ε∈[0,1-θ]. Hence ${}^{A}\mu_{s}\leq {}^{A}\mu_{s}^{\prime }$ for all *s* and ${}^{A}\mu_{s},{}^{A}\mu_{s}^{\prime }\in \left[0,1\right]$.

Then

$$\begin{array}{cc} {}^{A}\mu_{\mathrm{nc}}= & \frac{1}{\mathrm{Bc}(n)}\sum_{s=1}^{n-1}\sum_{t=s+1}^{n}{}^{A}\mu_{s}\cdot {}^{A}\mu_{t}=\frac{1}{\mathrm{Bc}(n)}\sum_{s=1}^{n-1}\sum_{t=s+1}^{n}\theta^{2}=\frac{1}{\mathrm{Bc}(n)}\theta^{2}\sum_{s=1}^{n-1}\left(\sum_{t=1}^{n}1-\sum_{t=1}^{s}1\right)\\ = & \frac{1}{\mathrm{Bc}(n)}\theta^{2}\sum_{s=1}^{n-1}\left(n-s\right)=\frac{1}{\frac{n}{2}\cdot \left(n-1\right)}\theta^{2}n\cdot \left(n-1\right)-\frac{n}{2}\cdot \left(n-1\right)=\theta^{2}. \end{array}$$

As no information is provided about further propositions, the remaining belief is assigned to the frame of discernment to satisfy Definition 5, i.e., ${}^{\Theta }\mu_{s}=1-{}^{A}\mu_{s}=1-\theta$ for all *s*. Thus

$${}^{\Theta }\mu_{\mathrm{nc}}={\left(1-\theta \right)}^{2},\;{}^{A}\mu_{c}=c\cdot \frac{1}{n}\sum_{s=1}^{n}{}^{A}\mu_{s}=\left(2a-2a^{2}\right)\cdot \theta =2a^{2}-2a^{3},$$

$$c=1-\sum_{i=1}^{o}{}^{A_{i}}\mu_{\mathrm{nc}}=1-\left({}^{A}\mu_{\mathrm{nc}}+{}^{\Theta }\mu_{\mathrm{nc}}\right)=1-\left(\theta^{2}+{\left(1-\theta \right)}^{2}\right)=1-\left(\theta^{2}+1-2a+\theta^{2}\right)=2a-2a^{2}.$$

Consequently,

(C1) $${}^{A}\mu ={}^{A}\mu_{\mathrm{nc}}+{}^{A}\mu_{c}=\theta^{2}+2a^{2}-2a^{3}=3a^{2}-2a^{3}.$$

Following the same argumentation for ${}^{A}\mu_{s}^{\prime }=\theta +\varepsilon$ for all *s* leads to

(C2) $${}^{A}\mu^{\prime }={}^{A}\mu_{\mathrm{nc}}^{\prime }+{}^{A}\mu_{c}^{\prime }=3{\left(\theta +\varepsilon \right)}^{2}-2{\left(\theta +\varepsilon \right)}^{3}.$$

If ${}^{A}\mu \leq {}^{A}\mu^{\prime }$, then $f\left(\theta \right)={}^{A}\mu^{\prime }-{}^{A}\mu \geq 0$:

$$\begin{array}{cc} f(\theta )= & 3{\left(\theta +\varepsilon \right)}^{2}-2{\left(\theta +\varepsilon \right)}^{3}-3a^{2}-2a^{3}=6\varepsilon \theta -6\varepsilon \theta^{2}-6\varepsilon^{2}\theta +3\varepsilon^{2}-2\varepsilon^{3}\\ = & -6\varepsilon \theta^{2}+6\left(\varepsilon -\varepsilon^{2}\right)\theta +3\varepsilon^{2}-2\varepsilon^{3}. \end{array}$$

In order to show f(θ)≥0 for all *θ* its roots and extrema are determined.

**Roots.**

$$f\left(\theta \right)=0\Leftrightarrow 0=-6\varepsilon \theta^{2}+6\left(\varepsilon -\varepsilon^{2}\right)\theta +3\varepsilon^{2}-2\varepsilon^{3}\Leftrightarrow 0=\theta^{2}+\left(\varepsilon -1\right)\theta -\frac{1}{2}\varepsilon +\frac{1}{3}\varepsilon^{2}.$$

Hence, f(θ)=0 for $\theta_{1/2}$ with

$$\begin{array}{cc} \theta_{1/2}= & -\frac{\varepsilon -1}{2}\pm \sqrt{{\left(\frac{\varepsilon -1}{2}\right)}^{2}-\left(\frac{1}{3}\varepsilon^{2}-\frac{1}{2}\varepsilon \right)}=\frac{1}{2}-\frac{\varepsilon }{2}\pm \sqrt{\frac{1}{4}-\frac{1}{2}\varepsilon +\frac{1}{4}\varepsilon^{2}-\frac{1}{3}\varepsilon^{2}+\frac{1}{2}\varepsilon }\\ = & \frac{1}{2}-\frac{\varepsilon }{2}\pm \sqrt{\frac{1}{4}-\frac{1}{12}\varepsilon^{2}}=\frac{1}{2}-\frac{\varepsilon }{2}\pm \frac{1}{2}\sqrt{\frac{3-\varepsilon^{2}}{3}}=\frac{1}{2}\left(1-\varepsilon \pm \sqrt{1-\frac{\varepsilon^{2}}{3}}\right) \end{array}$$

**Extrema.**

- Necessary criterion $\left(\frac{df}{d\theta }f\left(\theta \right)=0\right)$:
  $$0=-12\varepsilon \theta +6\left(\varepsilon -\varepsilon^{2}\right)\Leftrightarrow \theta =\frac{1}{2}\left(1-\varepsilon \right).$$
- Sufficient criterion $\left(\frac{d^{2}f}{d\theta^{2}}f\left(\theta \right)\neq 0\right)$:
  $$\frac{d^{2}f}{d\theta^{2}}f\left(\theta \right)=-12\varepsilon >0\;\mathrm{for}\;\varepsilon >0.$$

Consequently, f(θ) has a *maximum* at $\theta =\frac{1}{2}\left(1-\varepsilon \right)$ for ε>0.

The maximum is always between the roots of f(θ), therefore the quadratic function f(θ)≥0 for $\theta_{1}\leq \theta \leq \theta_{2}$. As $\theta_{1/2}$ depends on *ε*, the roots are evaluated for the minimal and maximal possible values of *ε*:

$\varepsilon =0:\;\theta_{1/2}=\frac{1}{2}\left(1\pm 1\right)\Rightarrow \theta_{1}=0,\theta_{2}=1$.
$\varepsilon =1-\theta :\;\theta_{1/2}=\frac{1}{2}\left(1-\left(1-\theta \right)\pm \sqrt{1-\frac{\theta^{2}}{3}}\right)$. From θ+ε≤1 follows that *θ* is further constrained by θ≤0.5, hence $\theta_{1}=-0.229,\theta_{2}\approx 0.738$.

That is, the roots are outside the domain of *θ*. Only in the case of ε=0, the roots are at the borders of *θ*’s domain. Hence, the quadratic function f(θ)≥0 for all θ∈[0,1] and consequently ${}^{A}\mu \leq {}^{A}\mu^{\prime }$. □
**Proof of Axiom 5**. Without loss of generality, let ${}^{\varepsilon }\mu_{1}={}^{A}\mu_{1}+\varepsilon$ and ${}^{\varepsilon }\mu_{s}={}^{A}\mu_{s}$ for all s≠1. As no information is provided about further propositions, the remaining belief is assigned to the frame of discernment to satisfy Definition 5. Thus, ${}^{\Theta }\mu_{1}=1-\left({}^{A}\mu_{1}+\varepsilon \right)$ and ${}^{\Theta }\mu_{s}=1-{}^{A}\mu_{s}$ for all s≠1. Then

$${}^{\varepsilon }\mu_{\mathrm{nc}}=\frac{1}{\mathrm{Bc}(n)}\sum_{s=1}^{n-1}\sum_{t=s+1}^{n}{}^{\varepsilon }\mu_{s}\cdot {}^{\varepsilon }\mu_{t}=\frac{1}{\mathrm{Bc}(n)}\left(\left({}^{A}\mu_{1}+\varepsilon \right)\cdot \sum_{t=2}^{n}{}^{A}\mu_{t}+\sum_{s=2}^{n-1}\sum_{t=s+1}^{n}{}^{A}\mu_{s}\cdot {}^{A}\mu_{t}\right),$$

$$\begin{array}{cc} {}^{\Theta }\mu_{\mathrm{nc}}= & \frac{1}{\mathrm{Bc}(n)}\sum_{s=1}^{n-1}\sum_{t=s+1}^{n}{}^{\Theta }\mu_{s}\cdot {}^{\Theta }\mu_{t}\\ = & \frac{1}{\mathrm{Bc}(n)}\left(\left(1-\left({}^{A}\mu_{1}+\varepsilon \right)\right)\cdot \sum_{t=2}^{n}\left(1-{}^{A}\mu_{t}\right)+\sum_{s=2}^{n-1}\sum_{t=s+1}^{n}\left(1-{}^{A}\mu_{s}\right)\cdot \left(1-{}^{A}\mu_{t}\right)\right), \end{array}$$

$${}^{\varepsilon }\mu_{c}=c\cdot \frac{1}{n}\sum_{s=1}^{n}{}^{A}\mu_{s}=c\cdot \frac{1}{n}\left(\left({}^{A}\mu_{1}+\varepsilon \right)+\sum_{s=2}^{n}{}^{A}\mu_{s}\right),$$

where $c=1-\sum_{i=1}^{o}{}^{A_{i}}\mu_{\mathrm{nc}}=1-\left({}^{\varepsilon }\mu_{\mathrm{nc}}+{}^{\Theta }\mu_{\mathrm{nc}}\right)$. Consequently, ${}^{\varepsilon }\mu ={}^{\varepsilon }\mu_{\mathrm{nc}}+{}^{\varepsilon }\mu_{c}$.

Next, ${}^{\varepsilon }\mu_{\mathrm{nc}}$, ${}^{\Theta }\mu_{\mathrm{nc}}$, and ${}^{\varepsilon }\mu_{c}$ are evaluated for $\lim_{\varepsilon \to 0}$:

$$\begin{array}{cc} \lim_{\varepsilon \to 0}{}^{\varepsilon }\mu_{\mathrm{nc}}= & \lim_{\varepsilon \to 0}\frac{1}{\mathrm{Bc}(n)}\left(\left({}^{A}\mu_{1}+\varepsilon \right)\cdot \sum_{t=2}^{n}{}^{A}\mu_{t}+\sum_{s=2}^{n-1}\sum_{t=s+1}^{n}{}^{A}\mu_{s}\cdot {}^{A}\mu_{t}\right)\\ = & \frac{1}{\mathrm{Bc}(n)}\left({}^{A}\mu_{1}\cdot \sum_{t=2}^{n}{}^{A}\mu_{t}+\sum_{s=2}^{n-1}\sum_{t=s+1}^{n}{}^{A}\mu_{s}\cdot {}^{A}\mu_{t}\right)=\frac{1}{\mathrm{Bc}(n)}\sum_{s=1}^{n-1}\sum_{t=s+1}^{n}{}^{A}\mu_{s}\cdot {}^{A}\mu_{t}={}^{A}\mu_{\mathrm{nc}}, \end{array}$$

$$\begin{array}{cc} \lim_{\varepsilon \to 0}{}^{\Theta }\mu_{\mathrm{nc}}= & \lim_{\varepsilon \to 0}\frac{1}{\mathrm{Bc}(n)}\left(\left(1-\left({}^{A}\mu_{1}+\varepsilon \right)\right)\cdot \sum_{t=2}^{n}\left(1-{}^{A}\mu_{t}\right)+\sum_{s=2}^{n-1}\sum_{t=s+1}^{n}\left(1-{}^{A}\mu_{s}\right)\cdot \left(1-{}^{A}\mu_{t}\right)\right)\\ = & \frac{1}{\mathrm{Bc}(n)}\left(\left(1-{}^{A}\mu_{1}\right)\cdot \sum_{t=2}^{n}\left(1-{}^{A}\mu_{t}\right)+\sum_{s=2}^{n-1}\sum_{t=s+1}^{n}\left(1-{}^{A}\mu_{s}\right)\cdot \left(1-{}^{A}\mu_{t}\right)\right)\\ = & \frac{1}{\mathrm{Bc}(n)}\sum_{s=1}^{n-1}\sum_{t=s+1}^{n}\left(1-{}^{A}\mu_{s}\right)\cdot \left(1-{}^{A}\mu_{t}\right), \end{array}$$

$$\lim_{\varepsilon \to 0}{}^{\varepsilon }\mu_{c}=\lim_{\varepsilon \to 0}c\cdot \frac{1}{n}\left(\left({}^{A}\mu_{1}+\varepsilon \right)+\sum_{s=2}^{n}{}^{A}\mu_{s}\right)=c\cdot \frac{1}{n}\left({}^{A}\mu_{1}+\sum_{s=2}^{n}{}^{A}\mu_{s}\right)=c\cdot \frac{1}{n}\sum_{s=1}^{n}{}^{A}\mu_{s}={}^{A}\mu_{c}.$$

This leads in summary to $\lim_{\varepsilon \to 0}{}^{\varepsilon }\mu ={}^{A}\mu_{\mathrm{nc}}+{}^{A}\mu_{c}={}^{A}\mu$. □
